# Catabolic Signaling Pathways, Atrogenes, and Ubiquitinated Proteins Are Regulated by the Nutritional Status in the Muscle of the Fine Flounder

**DOI:** 10.1371/journal.pone.0044256

**Published:** 2012-09-14

**Authors:** Eduardo N. Fuentes, Pamela Ruiz, Juan Antonio Valdes, Alfredo Molina

**Affiliations:** Laboratorio de Biotecnologia Molecular, Departmento de Ciencias Biologicas, Facultad de Biologia, Universidad Andres Bello, Santiago, Chile; University of Sydney, Australia

## Abstract

A description of the intracellular mechanisms that modulate skeletal muscle atrophy in early vertebrates is still lacking. In this context, we used the fine flounder, a unique and intriguing fish model, which exhibits remarkably slow growth due to low production of muscle-derived IGF-I, a key growth factor that has been widely acknowledged to prevent and revert muscle atrophy. Key components of the atrophy system were examined in this species using a detailed time-course of sampling points, including two contrasting nutritional periods. Under basal conditions high amounts of the atrogenes *MuRF-1* and *Atrogin-1* were observed. During fasting, the activation of the P38/MAPK and Akt/FoxO signaling pathways decreased; whereas, the activation of the IκBα/NFκB pathway increased. These changes in signal transduction activation were concomitant with a strong increase in *MuRF-1*, *Atrogin-1*, and protein ubiquitination. During short-term refeeding, the P38/MAPK and Akt/FoxO signaling pathways were strongly activated, whereas the activation of the IκBα/NFκB pathway decreased significantly. The expression of both atrogenes, as well as the ubiquitination of proteins, dropped significantly during the first hour of refeeding, indicating a strong anti-atrophic condition during the onset of refeeding. During long-term refeeding, Akt remained activated at higher than basal levels until the end of refeeding, and Atrogin-1 expression remained significantly lower during this period. This study shows that the components of the atrophy system in skeletal muscle appeared early in the evolution of vertebrates and some mechanisms have been conserved, whereas others have not. These results represent an important achievement for the area of fish muscle physiology, showing an integrative view of the atrophy system in a non-mammalian species and contributing to novel insights on the molecular basis of muscle growth regulation in earlier vertebrates.

## Introduction

The maintenance of skeletal muscle mass is a complex and controlled process that is largely influenced by the nutritional and physiological state of the animal. This dynamic process is regulated by a balance between protein synthesis and protein degradation; however, when rates of protein degradation exceed rates of protein synthesis, then muscle mass is lost, leading to atrophy of this tissue [1]. Muscle atrophy occurs as a consequence of denervation, injury, joint immobilization, glucocorticoid treatment, sepsis, cancer, and aging [1]. Food deprivation and undernourishment are also two main conditions that promote muscle atrophy; thus, highlighting that nutritional status has a major role in skeletal muscle mass regulation [2], [3]. The major route that increases overall rates of protein degradation during muscle atrophy is the ubiquitin–proteasome pathway [4], [5]. Polyubiquitination of proteins is a multiple-step process that requires ATP and the participation of three components in the formation of the ubiquitin-protein complexes, the ubiquitin-activating enzyme (E1), a ubiquitin-conjugating enzyme (E2), and a ubiquitin-ligase (E3) [6], [7], [8], in order to covalently attach multiple ubiquitin molecules to the protein substrate [6]. Subsequently, these tagged proteins are recognized and degraded by the 26S proteasome, resulting in short peptides [8]. Particularly, the ubiquitin-ligases are a family of key enzymes responsible for transferring an activated ubiquitin molecule to a targeted protein, subsequently marking the protein for proteasomal degradation [8]. Indeed, an increase in the capacity for protein degradation via the proteasome is dependent on an increase in ubiquitin-ligase expression [9]. Numerous ubiquitin-ligases have been identified; however, differential expression screening studies, originally planned to detect high-fidelity markers of muscle atrophy, led to the discovery of two genes that encode ubiquitin-ligases, MuRF-1 (Muscle Ring Finger protein-1) and Atrogin-1 (also called Muscle Atrophy F-box (MAFbx) [10], which have been shown to be upregulated in several models of skeletal muscle atrophy, validating them as reliable markers of atrophy [11], [12], [13].

The transcriptional regulation of these ubiquitin-ligases, also named atrogenes, during catabolic-atrophic processes in skeletal muscle has been linked to the activation of different signaling pathways, such as the mitogen-activated protein kinases (MAPKs), specifically the P38; the protein kinase B (Akt)/forkhead box O (FoxO); and the inhibitor of kappa, alpha (IκBα)/nuclear factor kappa B (NFκB) [13]. The Akt/FoxO signal transduction is the only signaling pathway able to regulate the expression of both atrogenes, upregulating their transcription when Akt activation decreases and reducing the phosphorylation FoxO1/3 transcription factors, promoting their nuclear translocation [9]. On the other hand, the other two signaling pathways independently stimulate the expression of these atrogenes, showing functional separation of the expression of *MuRF-1* and *Atrogin-1* [13]. The activation of the IKKβ/IκBα/NFκB signaling pathway promotes the upregulation of *MuRF-1*, causing severe muscle wasting and atrophy, a phenomenon that is reverted when this signaling pathway is blocked [14]. In general terms, NFκB in an inactivated state is located in the cytosol of muscle cells and is associated with the inhibitory protein IκBα. Upon stimulation, IκBα is phosphorylated by the IκB kinase (IKK), ubiquinated, and degraded, thus dissociating the complex formed by IκBα and NFκB and allowing the translocation into the nucleus of NFκB, ultimately promoting the transcription of *MuRF-1* [13], [14]. Conversely, *Atrogin-1* upregulation is promoted by the P38/MAPK signaling pathway [15], through FoxO4 [16], and the Akt/FoxO1/3 signaling pathway [9], [17].

Knowledge concerning the molecular mechanisms that modulate muscle atrophy in fish has been scarce and limited, mainly focusing on the cloning and evaluation of expression patterns of both atrogenes [18]–[22]. A few studies have gone further in the understanding of muscle atrophy in teleosts by using transcriptomic and proteomic approaches [23]–[25]. However, assessment of the intracellular pathways modulating muscle catabolism, as well as the main components of the atrophy system, has not been performed (e.g. P38/MAPK, Akt/FoxO, and IκBα/NFκB signaling pathways, MuRF-1 and Atrogin-1, ubiquitin proteasome-dependent proteolysis). Hence, the study of this system represents important progress for the area of fish muscle physiology, especially due to the unique features displayed by this tissue in this group of vertebrates, which include muscle atrophy as part of a natural seasonal cycle, due to changes in food availability, and indeterminate growth by hyperplasia and hypertrophy [26]–[29].

The aim of this study was to assess the atrophy system in the skeletal muscle of a teleost fish using a detailed time-course of sampling points including two contrasting nutritional periods: an extended catabolic period of fasting, leading to muscle atrophy, followed by an anabolic period of refeeding, leading to compensatory muscle hypertrophy. We used the fine flounder (*Paralichthys adspersus*) as a model, which is a unique and intriguing flatfish species that exhibits natural growth deficiency [30], [31]. The responsible mechanism for this impairment in growth has been associated to an inherent growth hormone (GH) resistance in skeletal muscle. Thus, this fish represents a unique model of low basal production of muscle-derived insulin-like growth factor-I (IGF-I) [31], which is a key molecule that induces muscle hypertrophy via the Akt/mTOR pathway, concomitant with preventing atrophy [10], [13], [32], by downregulating the expression of *MuRF-1* and *Atrogin-1* [9], [33], [34] acting through the PI3K/Akt/FoxO pathway [9], [33].

## Materials and Methods

### Ethics Statement

The study adhered to animal welfare procedures and was approved by the bioethical committees of the Universidad Andres Bello and the National Commission for Scientific and Technological Research of the Chilean government.

### Fish Husbandry, Experimental Design, and Sampling

Three-year old, sexually immature juvenile fine flounder (*Paralichthys adspersus*), with an average weight of 300±10 g, were maintained under natural temperature (12°C±3) and photoperiod (13 hrs light: 11 hrs dark) conditions corresponding to the geographic location (33°13′S; 71°38′W) of the Centro de Investigacion Marina de Quintay (CIMARQ) in the southern hemisphere during the spring season of 2009 (October-December). Fish were hand fed once daily with 9-mm commercial pellet containing 45% protein, 22% lipids, 16% carbohydrates, 1% crude fiber, 7% ashes, and 10% humidity (Skretting, Puerto Montt). Fish were randomly divided into two circular (ø 3.85 m) fiberglass tanks (150 fish per tank). The tanks were 75 cm in height with a water column of 30 cm, and a water turn-over of 15.4 L min^−1^ (one water clearance per hour).

The experimental design consisted in fish acclimatized for two weeks under satiety feeding conditions. At the start of the experiment (week 0), food was withheld from fish for three weeks, inducing a nutritional catabolic state of fasting. Then, fish were subjected to a four week satiety refeeding period, which returned the fish to an anabolic state. Body weight and length were measured to show the effects of nutritionally-induced catabolic/anabolic states on growth performance [31].

Samples were obtained weekly over the trial (i.e. fasting and refeeding) with the purpose of studying long-term changes in the atrophy system of skeletal muscle. Also, in order to study short-term changes of this system during the refeeding period, fish were sampled at 2, 4, and 24 hours after the first meal supply following the three weeks of fasting. For each sampling point, three individuals were sampled (n = 3). Sampling was performed under anesthesia (3-aminobenzoic acid ethyl ester, 100 mg/l^−1^), and white-fast myotomal muscle was subsequently collected. Tissue was frozen immediately in liquid nitrogen and stored at −80°C until processing.

### Western Blotting

Total protein were extracted and prepared according to Fuentes et al., [31], [35]. Protein concentration was determined by a Pierce® BCA Protein Assay Kit (Thermo Scientific, IL, USA).

Western blot assays were performed according to Fuentes et al., [31], [35]. Briefly, proteins were transferred to polyvinylidene diflouride membranes (PVDF) (Millipore, Bedford, MA, USA) and blocked for 1 h at room temperature in 2% ECL Advance™ blocking agent (GE Healthcare, Buckinghamshire, UK) dissolved in tris-buffered saline (TBS 1×). Primary antibody incubations (phosphorylated (p) P38, Akt, FoxO, IκBα and total Ubiquitin) were performed at 4°C overnight. All antibodies were commercial antibodies purchased from Cell Signaling (Beverly, MA). Details of the antibodies are found in Table 1. Membranes were visualized by a high sensitivity enhanced chemiluminescence kit, the ECL Advance™ Western Blotting Detection Kit (GE Healthcare, Buckinghamshire, UK), according to the manufacturer’s instructions. Subsequently, membranes were stripped and blotted for total P38, Akt, FoxO, and IκBα antibodies respectively (Table 1), therefore obtaining the ratio between the phosphorylated protein with the total protein. For ubiquitinated protein assessment, they were normalized respectively by comparison with *Coomassie* blue (Cb) staining of total protein. The obtained films were then scanned, and densitometric analysis of the bands was performed with the Image J program (National Institute of Health, USA). Western blots were carried out on three individual samples (N = 3 per sampling time-point), showing a representative blot film. All graphs for long-term changes during fasting and refeeding are expressed as a fold change over the basal levels found at the beginning of the trial (week 0), whereas for short-term refeeding, graphs are expressed as a fold change over levels found at the end of fasting (week 3).

**Table 1:** Antibody types and features used for the detection of components of the atrophy system.

Antibodies	Dilution	Molecular Weight (kDa)	Brand	Catalog Number
P38 (a)	1∶5,000	43	Cell Signaling	9212
pP38 (a)	1∶5,000	43	Cell Signaling	9211
IkBa (b)	1∶1,000	39	Cell Signaling	4814
pIkBa (c)	1∶1,000	40	Cell Signaling	2859
Akt (a)	1∶5,000	60	Cell Signaling	9272
pAkt (a)	1∶5,000	60	Cell Signaling	9271
FoxO1 (c)	1∶1,000	82	Cell Signaling	2880
pFoxO1/3 (c)	1∶1,000	82, 95	Cell Signaling	9464
Ubiquitin (a)	1∶5,000		Cell Signaling	3993
IgG	1∶10,000 (a)		Cell Signaling	7074
	1∶2,000 (c)			
IgG*	1∶2,000 (b)		Cell Signaling	7076

Similar letters (a or b) indicate the dilution used for primary and secondary antibodies.

**Figure 1:**
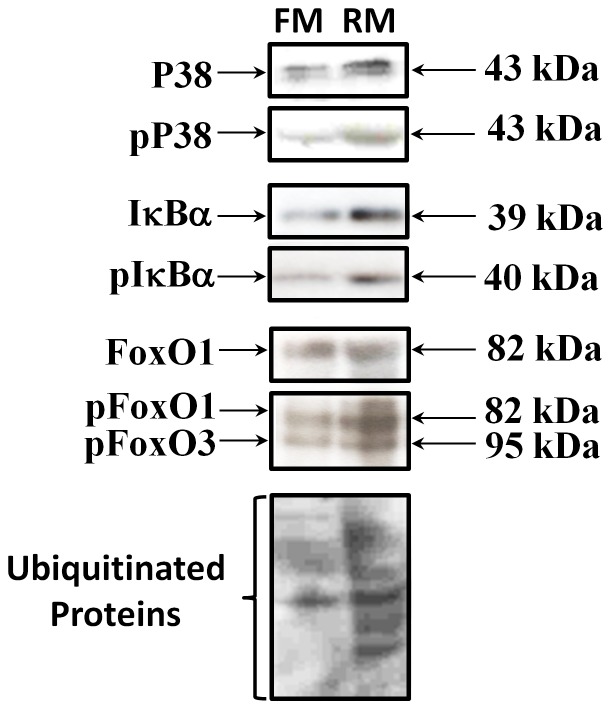
Comparative western blot between fine flounder skeletal muscle (FM) and primary culture of rat skeletal muscle (RM), showing all the antibodies used in the present study.

In order to validate and confirm that antibodies developed against mammalian epitopes cross-react with samples of skeletal muscle of the fine flounder, comparative western blots were performed using rat myosatellite cell primary cultures as a control, indicating that all antibodies cross-react with their orthologs in the flounder (Fig. 1). Akt and pAkt antibodies were previously validated by Fuentes et al., [35].

### RNA Extraction and cDNA Synthesis

Total RNA was extracted from skeletal muscle using the RNeasy Mini Kit (Qiagen, Austin, TX, USA) following manufacturer’s recommendations. RNA was quantified using NanoDrop technology with the Epoch Multi-Volume Spectrophotometer System (BioTek, Winooski, VT, USA). Assessment of RNA quality was performed by electrophoresis on a 1.2% formaldehyde agarose gel containing ethidium bromide. Only RNAs with an A260/280 ratio between 1.9 and 2.1 were used for cDNA synthesis. Residual genomic DNA was removed using the genomic DNA wipeout buffer included in the Quantitect® reverse transcription kit (Qiagen, Austin, TX, USA). Subsequently, 800 ng of RNA were reverse transcribed into cDNA for 30 min at 42°C using the manufacturer’s recommendations.

### Isolation and Cloning of Atrogenes

Once high quality cDNA was obtained from muscle as previously described, *MuRF-1* and *Atrogin-1* were isolated and deposited in the GeneBank, GeneBank Accession Numbers are indicated in Table 2. Primers used for obtaining the sequences of atrogenes were designed by multiple alignments of sequences from fish species using ClustalW (Table 2). PCR was performed using 1µL cDNA template, 5 µL of PCR buffer 10X, 200 µM of each dNTP, 500 nM of each primers, 0.3 µL of Taq DNA polymerase (12 U/µL) (Promega, Madison, WI, USA), and RNAse-free water to a final volume of 50 µl. Thermal cycling conditions were the following: initial activation of 10 min at 95°C, followed by 40 cycles of 30 s at 95°C; 30 s at 55°C (*MuRF-1*) or 57°C (*Atrogin-1*); and 30 s at 72°C with a final extension of 10 min at 72°C.

**Table 2:** Primer sequences for cloning and qPCR assay of MuRF-1 and Atrogin-1 in the fine flounder.

Gene	Primer	Secuence (5′–3′)	Size (pb)	E(%)	Accession number
*MuRF-1*	Forward cloning	CTGGAGGAACGTAAGGGC			JN801157
	Reverse cloning	TCCATGTTCTCGAAGCCA			
	Forward qPCR	TCTGGTGTCTTCCGAT	172	99.3	
	Reverse qPCR	TTGGCTAACGCAATAGA			
*Atrogin-1*	Forward cloning	GACAACATTCAGATCAACAGGC			JN801155
	Reverse cloning	CCAGAAGAGGATGTGGCAGT			
	Forward qPCR	TGACTCTGACCGAACTGCC	226	100.3	
	Reverse qPCR	AAGTGGTGCTGGCAGAGTTT			

Amplicon size (bp), qPCR efficiencies (E(%)), and GeneBank accession number are also shown.

Cloning procedure was carried out using the TOPO TA Cloning® system (Invitrogen, Carlsbad, CA, USA) using manufacturer’s recommendations. In short, PCR products were ligated into the T/A pCR4-TOPO vector (Invitrogen, Carlsbad, CA, USA), and subsequently, One Shot® TOP10 competent *E. coli* (Invitrogen, Carlsbad, CA, USA) were transformed with the vector. Individual colonies were cultured, and plasmids were isolated, purified using the QIAGEN® Plasmid Purification (QIAGEN), and subsequently sequenced.

### Quantitative Real-time PCR (qPCR)

All primer design for qPCR was based on partial sequences previously obtained. In order to get high quality primers and avoid secondary structures (i.e. hairpins, homo- and cross-dimers), Amplifx 1.5.4 (http://ifrjr.nord.univ-mrs.fr/AmplifX-Home-page) and Primer 3 (http://frodo.wi.mit.edu/primer3/) programs were used in order to obtain candidate primers. Subsequently, primer pairs were validated using the NetPrimer software (http://www.premierbiosoft.com/netprimer/). Primers used for qPCR are listed in Table 2.

All the procedures were carried out according to the protocol outlined by Bower et al., [36] with minor modifications. All qPCR assays were carried out to complying with the Minimum Information for Publication of Quantitative Real-Time PCR experiments MIQE guidelines [37].

Total RNA extraction and cDNA synthesis from skeletal muscle of all sampling points were performed as described above. Quantitative PCR (qPCR) was performed using the Stratagene MX3005P qPCR system (Stratagene). Each qPCR reaction mixture contained 7.5 µL Brilliant II SYBR green master mix (Agilent Technologies, SC, USA), 6 µL cDNA (40-fold dilution), 250 mM of each primer, 5 µM ROX, and RNAse-free water to a final volume of 15 µL. Amplifications were performed in triplicate with the following thermal cycling conditions: 95°C for 10 min, followed by 35 cycles of 30 s at 95°C, 30 s at 54°C (*MuRF-1*) or 56°C (*Atrogin-1*), and 30 s at 72°C. In order to confirm the presence of a single PCR product, dissociation curve analysis of the PCR products was performed. Products were also evaluated by electrophoresis on a 1.5% agarose gel to confirm that a single product was amplified. With the purpose of estimating the efficiency of the assays, 2-fold dilutions series were created. Efficiency values were estimated from the slope of the curve following the equation: efficiency E = 10(−1/slope)^ −1^ (Table 2). Control reactions included a no template control (NTC) and a control without reverse transcriptase (-RT). An interplate calibrator (IPC) was used in all runs in order to correct plate to plate variation. Moreover, in order to corroborate the results, qPCR experiments were independently performed two times. qPCRs were carried out on three individual samples (N = 3 per sampling time-point). For long-term observations (i.e. fasting and refeeding), graphs are expressed as a fold change over the basal levels found at the beginning of the trial (week 0). For short-term observations (i.e. short-term refeeding), graphs are expressed as a fold change over levels found at the end of fasting (week 3). For atrogenes’ mRNA contents, graphs are represented as arbitrary units. For gene expression normalization of atrogenes we used the geometric average of the combination of the two most stable reference genes, 40S ribosomal protein S30 (*Fau*) and rRNA 18S (*18S*) [Fuentes EN, Safian D, Valdes JA & Molina A. 2012, unpublished results]. These genes were obtained by using the geNorm program, which obtained the normalization factor and subsequent relative expression levels of both atrogenes [38].

### Heat Map Summary of Hierarchical Clustering of Atrophy System Data

In order to establish relationships among all the components of the atrophy system, a heat map summary and hierarchical clustering analysis were performed using Permutmatrix [39]. All data points obtained from the activation of the signaling pathways, relative expression profiles of both atrogenes, and ubiquitinated proteins throughout the time-course events in different feeding statuses were incorporated into the Permutmatrix software. Clustering and seriation were based on Pearson’s correlation coefficient of z-score normalized abundance values (scaled from 0 to 1). McQuittýs method was used as a hierarchical clustering. The Permutmatrix program was also used to illustrate signaling pathway activation, relative expression profiles, and the amount of ubiquitinated proteins throughout the time-course events in different feeding statuses [39].

### Statistical Analyses

Statistical analyses used for studying differences in gene expression were based on an advanced linear model. This model was the general linear model (GLM), and was followed by Tukey’s analyses as post-test. Also, correlations between different parameters (i.e. molecules of the atrophy system) were assessed through multiple linear regressions, obtaining the coefficient of determination (R^2^) and the P-value. All statistical analyses were performed using the STATISTICA 7 software (Tulsa, OK, USA).

## Results

### The Atrophy Signaling Pathways (P38/MAPK, Akt/FoxO, and IκBα/NFκB) in the Skeletal Muscle of the Fine Flounder During Nutritionally-induced Catabolic and Anabolic Periods

**Figure 2:**
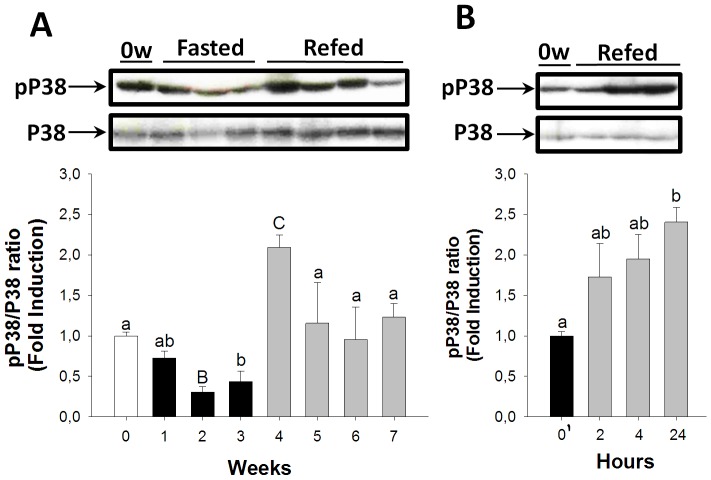
Fasting and refeeding effects on the P38/MAPK signaling pathway activation in the skeletal muscle. pP38/P38 ratios during long-term fasting and refeeding (A) and short-term refeeding (B). White, black, and grey bars represent periods of feeding, fasting, and refeeding, respectively. A probability level of P<0.05 (lower case letters) and P<0.01 (upper case letters) was used to indicate statistical significances. Results are expressed as means±SEM (n = 3). Different letters indicate significant differences among sampling points of each group, respectively. Abbreviations: 0′ = zero hour of short-term refeeding corresponding to the end of fasting period (week 3).

The P38/MAPK, Akt/FoxO, and IκBα/NFκB signal transduction pathways were assessed during fasting and refeeding in the teleost species the fine flounder in order to study the activation dynamics of the main signal transductions associated with muscle atrophy. The P38/MAPK activation pathway displayed a steady decrease during fasting, becoming statistically significant after two weeks (Fig. 2A). During long-term refeeding, this signaling pathway returned to basal levels of activation, except during the first week of refeeding where P38 significantly increased (Fig. 2A). During short-term refeeding after the three weeks of fasting, P38/MAPK signaling pathway activation increased steadily and showed significant differences at 24 hours (Fig. 2B).

**Figure 3:**
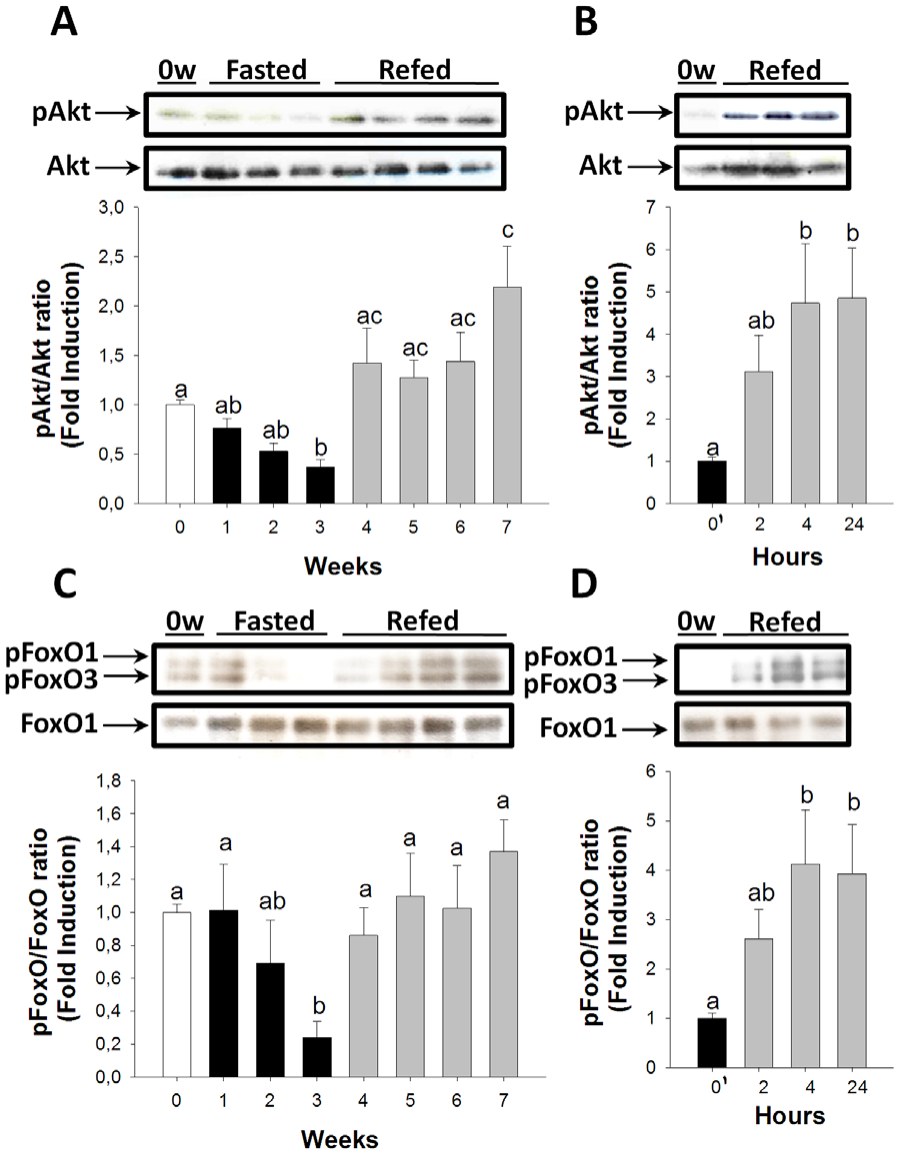
Fasting and refeeding effects on Akt/FoxO signaling pathway activation in the skeletal muscle. pAkt/Akt ratios during long-term fasting and refeeding (A) and short-term refeeding (B). pFoxO/FoxO ratios during long-term fasting and refeeding (C) and short-term refeeding (D). White, black, and grey bars represent periods of feeding, fasting, and refeeding, respectively. A probability level of P<0.05 (lower case letters) and P<0.01 (upper case letters) was used to indicate statistical significances. Results are expressed as means±SEM (n = 3). Different letters indicate significant differences among sampling points of each group, respectively. Abbreviations: 0′ = zero hour of short-term refeeding corresponding to the end of fasting period (week 3).

Akt/FoxO signaling pathway activation also decreased during fasting and became significantly diminished after three weeks (Fig. 3A, 3C). This pathway returned to basal levels during long-term refeeding; however, it was Akt, not FOXO, activation that increased significantly at the end of long-term refeeding (Fig. 3A, 3C). During short-term refeeding after the three weeks of fasting, Akt/FoxO signaling pathway activation increased steadily, showing significant differences at 4 and 24 hours (Fig. 3B, 3D).

**Figure 4:**
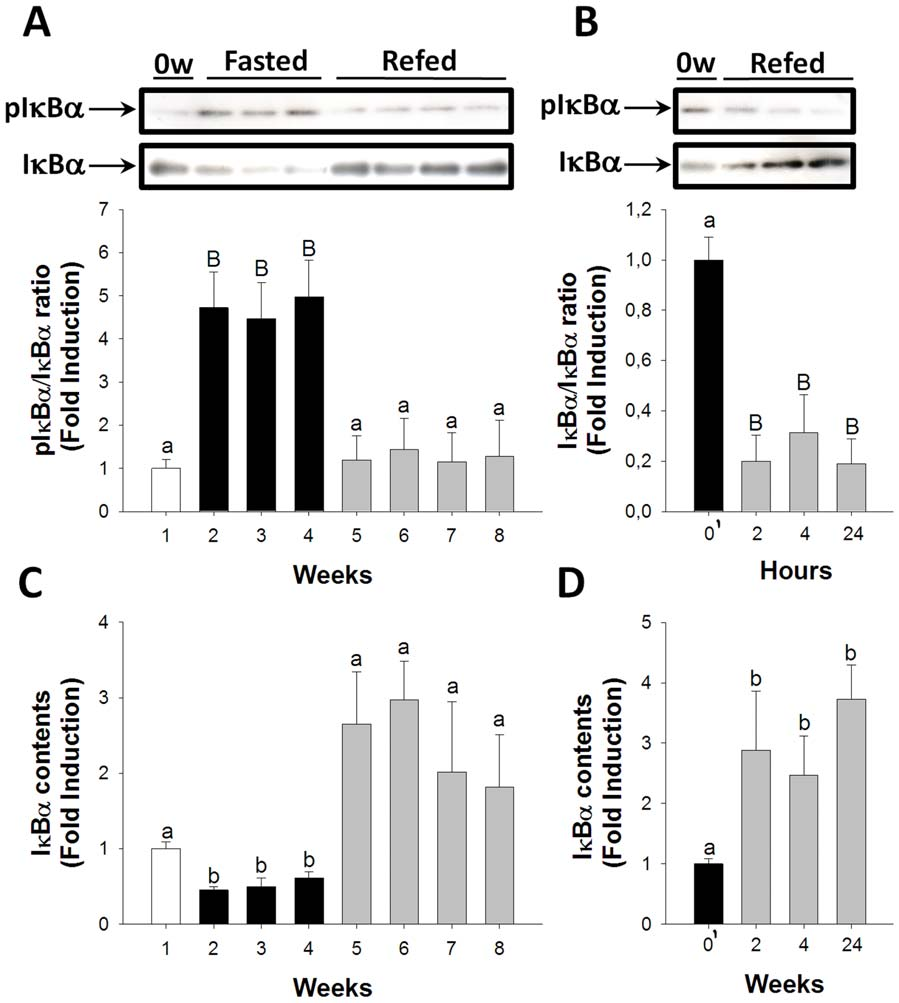
Fasting and refeeding effects on IκBα/NFκB signaling pathway activation in skeletal muscle. pIκBα/IκBα ratios during long-term fasting and refeeding (A) and short-term refeeding (B). IκBα degradation during long-term fasting and refeeding (C) and short-term refeeding (D). White, black, and grey bars represent periods of feeding, fasting, and refeeding, respectively. A probability level of P<0.05 (lower case letters) and P<0.01 (upper case letters) was used to indicate statistical significances. Results are expressed as means±SEM (n = 3). Different letters indicate significant differences among sampling points of each group, respectively. Abbreviations: 0′ = zero hour of short-term refeeding corresponding to the end of fasting period (week 3).

On the other hand, activation of the IκBα/NFκB signaling pathway rapidly increased during fasting, with significant differences found in the activation of IκBα from the first until the last week of fasting (Fig. 4A). IκBα activation returned to basal levels during long-term refeeding (Fig. 4A). Short-term refeeding observations showed that IκBα activation decreased rapidly, showing significant differences as early as two hours after refeeding (Fig. 4B). A significant decrease from the first until the third week of fasting was observed when assessing total IκBα protein contents, indicating degradation of IκBα (Fig. 4C). IκBα protein contents returned to basal levels during long-term refeeding. During short-term refeeding total IκBα protein contents increased, reflecting a decrease in the degradation of this protein (Fig. 4D).

### Expression of Atrogenes in Skeletal Muscle during Nutritionally-induced Catabolic and Anabolic Periods

**Figure 5:**
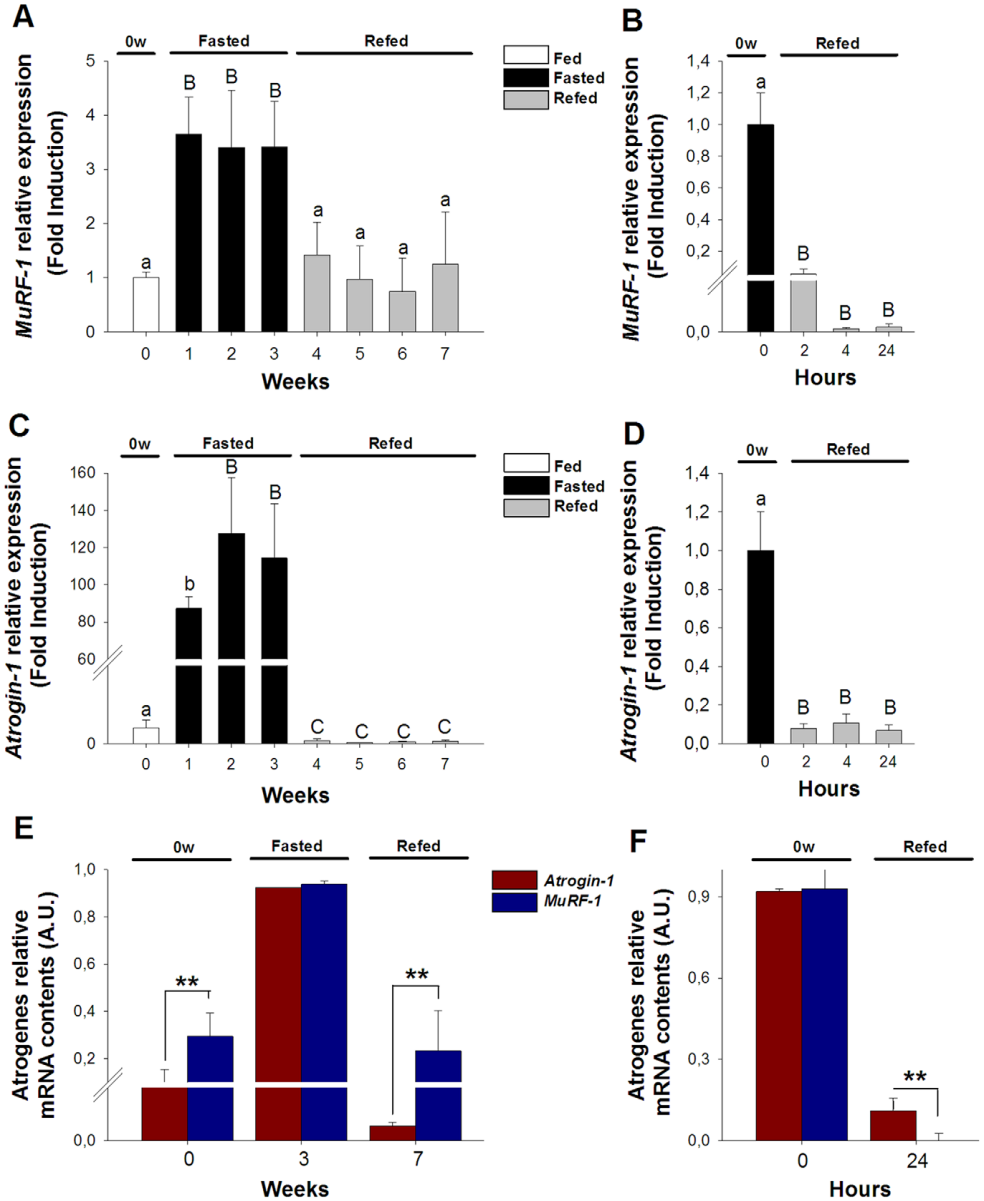
Transcriptional regulation of *MuRF-1* and *Atrogin-1* in the skeletal muscle during long-term fasting and refeeding and short-term refeeding. *MuRF-1* relative expression during long-term fasting and refeeding (A) and short-term refeeding (B). Atrogin-1 relative expression during long-term fasting and refeeding (C) and short-term refeeding (D). Relative mRNA content comparison between *MuRF-1* and *Atrogin-1* during long-term fasting and refeeding (E) and short-term refeeding (F). White, black and grey bars represent periods of feeding, fasting and refeeding, respectively. Red and blue bars represent *Atrogin-1* and *MuRF-1* expression respectively. A probability level of P<0.05 (lower case letters) and P<0.01 (by upper case letters) was used to indicate statistical significances. Results are expressed as means±SEM (n = 3). Different letters indicate significant differences among sampling points of each group, respectively. Abbreviations: 0′ = zero hour of short-term refeeding corresponding to the end of fasting period (week 3).

In order to study the dynamic of the two main atrogenes involved in muscle atrophy, which are direct downstream targets of the signaling pathways assessed above, the expression of *MuRF-1* and *Atrogin-1* was studied. During fasting, *MuRF-1* expression in muscle increased rapidly, with almost four-fold higher mRNA levels than basal levels from the first to the third week (Fig. 5A). During long-term refeeding, *MuRF-1* expression was restored to basal levels (Fig. 5A). During short-term refeeding, the most drastic changes were observed. In particular, *MuRF-1* decreased abruptly during this period, with a difference of more than one thousand-fold lower mRNA levels after 4 to 24 hours of refeeding than at the immediate end of fasting (0′ hours) (Fig. 5B).

Like *MuRF-1*, *Atrogin-1* also increased during fasting; nevertheless, changes in the expression of this atrogene were more radical and displayed a steady increase. More than one hundred-fold higher mRNA levels of *Atrogin-1,* as compared to basal conditions, were found at the end of fasting (Fig. 5C). During long-term refeeding, *Atrogin-1* decreased to lower than basal levels in all sampling points (Fig. 5C). During short-term refeeding, *Atrogin-1* also showed a drastic decrease, displaying more than twenty-fold lower mRNA levels during all sampling points than at the immediate end of fasting (0′ hours) (Fig. 5D).

By assessing specific levels of expression for both atrogenes, it is possible to observe that they were differentially expressed at basal levels, with higher levels of mRNA contents for *MuRF-1* than for *Atrogin-1* (*MuRF-1* was expressed five-fold more than *Atrogin-1*) (Fig. 5E). During fasting, both *MuRF-1* and *Atrogin-1* mRNA levels increased significantly, reaching similar levels (Fig. 5E, 5F). During long-term refeeding, *MuRF-1* was more expressed than *Atrogin-1*, and a similar trend to basal levels was found; however, the difference between the atrogenes at the end of refeeding was ninety six-fold (Fig. 5E). During short-term refeeding, both atrogenes dropped significantly; however, *MuRF-1* decreased even further than *Atrogin-1* mRNA levels, with a seventy three-fold lower expression of *MuRF-1* than of *Atrogin-1* (Fig. 5F).

### Ubiquitination of Total Protein in Skeletal Muscle during Nutritionally-induced Catabolic and Anabolic Periods

**Figure 6:**
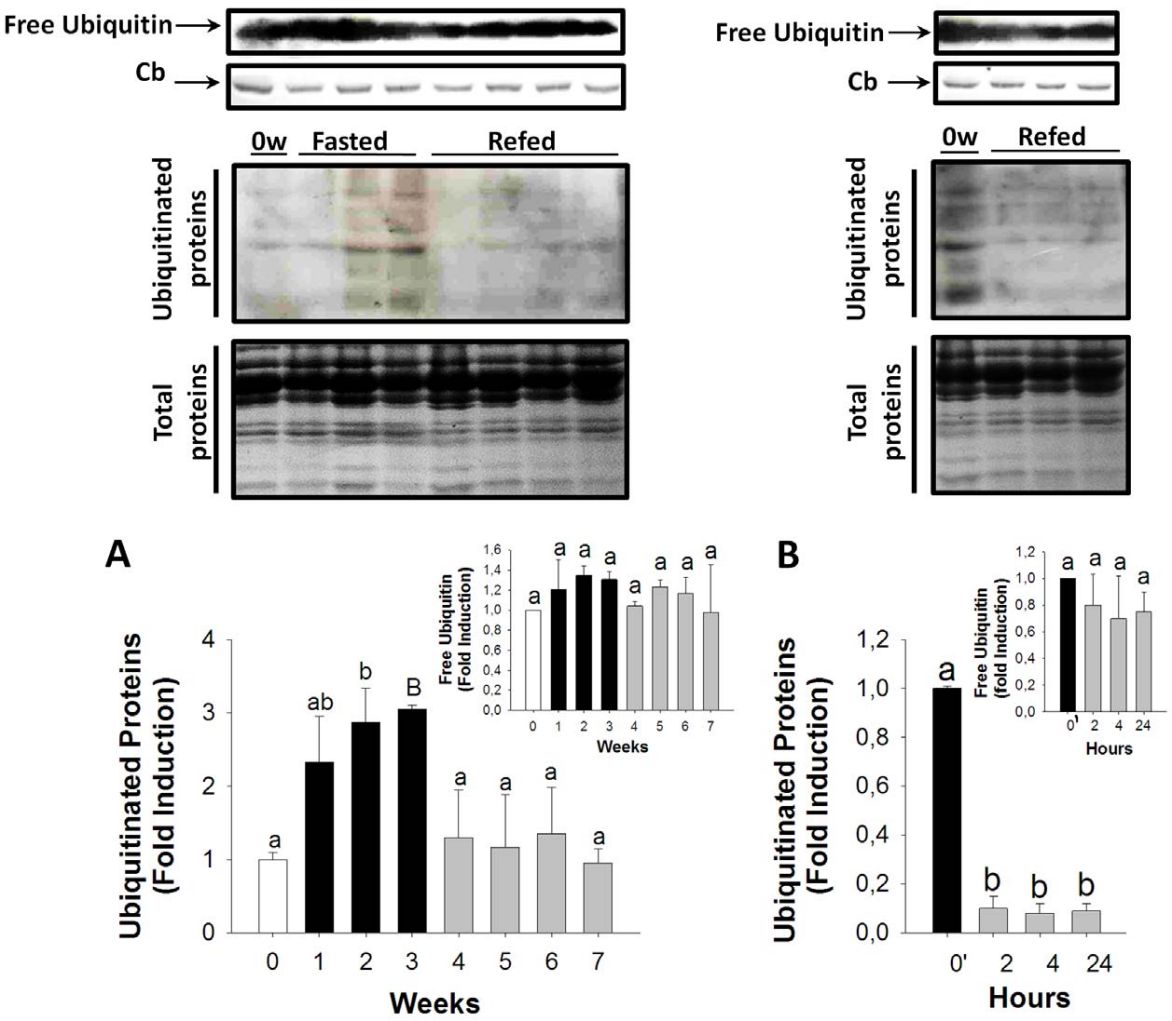
Ubiquitination of proteins in the skeletal muscle during long-term fasting and refeeding and short-term refeeding. Ubiquitinated protein during long-term fasting and refeeding (A) and short-term refeeding (B). Inserts in A and B show the amount of free ubiquitin during the trial. Percentage of ubiquitinated proteins during long-term fasting and refeeding (C) and short-term refeeding (D). White, black and grey bars represent periods of feeding, fasting and refeeding, respectively. A probability level of P<0.05 (lower case letters) and P<0.01 (by upper case letters) was used to indicate statistical significances. Results are expressed as means±SEM (n = 3). Different letters indicate significant differences among sampling points of each group, respectively. Abbreviations: Cb = *Coomassie* blue staining; 0′ = zero hour of short-term refeeding corresponding to the end of fasting period (week 3).

During fasting, ubiquitinated proteins increased steadily and were significant at the end of fasting (three-fold higher levels than 0 week) (Fig. 6A). During long-term refeeding, ubiquitinated proteins returned to basal levels (Fig. 6A). On the other hand, short-term refeeding triggered a rapid decrease in ubiquitinated proteins, finding significantly lower levels as early as two hours post-refeeding, and which were maintained until the first 24 hours (Fig. 6B). The amount of free ubiquitin did not change significantly during the trial (Inserts in Fig. 6A and 6B).

### Integration of Atrophy System Components in Skeletal Muscle During Nutritionally-induced Catabolic and Anabolic Periods

**Figure 7:**
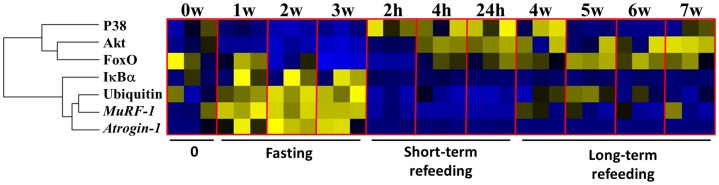
Summary of the atrophy system in the skeletal muscle of the fine flounder. Heat map summary and hierarchical clustering of the components of the atrophy system in the skeletal muscle of the fine flounder along the trial (A). In the heat map, the blue and yellow colors respectively indicate a decrease and increase in any of the components of the atrophy system.

Hierarchical clustering of the atrophy system throughout the trial showed two clades. The first clade showed a close relation and co-variation of Akt and FoxO activation, which was subsequently clustered with P38 activation. The second clade clustered ubiquitinated proteins with *MuRF-1* expression and, subsequently, *Atrogin-1* expression. This clade was clustered with IκBα activation (Fig. 7A).

## Discussion

The balance between muscle loss and growth, which determines skeletal muscle size, is dynamically controlled by specific signaling pathways that will either trigger an increase in protein degradation to diminish muscle mass (atrophy) or an increase in protein synthesis to stimulate muscle fiber growth (hypertrophy). The present study simulates this by withholding food from fine flounder for an extended period (3 weeks), inducing a catabolic period of muscle atrophy that is followed by a sustained nutritionally favorable period (4 weeks), which then induces an anabolic stage of compensatory muscle hypertrophy.

### Signaling Pathways Involved in Muscle Atrophy Linked to Atrogenes Expression and Protein Ubiquitination in Muscle of the Fine Flounder

By evaluating the transcriptional regulation of *MuRF-1* and *Atrogin-1*, a quite dissimilar pattern is observed via different temporal changes and abundances of these atrogenes. *MuRF-1 *mRNA is more abundant than *Atrogin-1* mRNA, with equivalent expression levels of structural muscle proteins and house-keeping genes (*MuRF-1* (Ct = 21), β-tubulin (Ct = 25), β-actin (Ct = 21), Fau (Ct = 23)) [Fuentes EN, Safian D, Valdes JA, Molina A. 2012. unpublished data]. During fasting, *MuRF-1* expression rapidly increases while *Atrogin-1* displays a steadier and more dramatic increase, and both reach similar levels at the end of this period. During refeeding, mRNA levels of both atrogenes drop rapidly and radically. However, *MuRF-1* shows an intense transcriptional regulation during only short-term refeeding, whereas *Atrogin-1* shows this during both long-and short-term refeeding. These results strongly suggest that different transcriptional controls are influencing these atrogenes.

Merging previous information on the expression of both atrogenes with the activation information of different signaling pathways and of the ubiquitination of proteins, an important relationship and temporal synchronicity among some components of the atrophy system is observed; however, not for all of them.

P38/MAPK activation decreases during fasting and increases during refeeding. The activation of this pathway does not show a similar trend and synchronicity with any of the atrogenes. In mammals, the P38/MAPK pathway was initially described as controlling cellular responses to stressors including pro-inflammatory cytokines, lipopolysaccharides (LPS), and UV light [40]. Particularly, this was corroborated in skeletal muscle by showing that the tumor necrosis factor- alpha (TNF-α), a pro-inflammatory cytokine, stimulates the expression of *Atrogin-1* via the P38/MAPK pathway [15]. Other more recent and detailed approaches have also supported this notion [41], [42]. On the other hand, studies have shown a positive role of the P38/MAPK pathway during myogenesis through the promotion of differentiation [40], [43] and inhibition of proliferation [43]. This dual role played by this signaling pathway is initiated and promoted by IGF-I [43]. Interestingly, P38 activation shows a similar pattern as that in both endocrine (circulatory/systemic, liver-derived) and autocrine/paracrine (local, muscle-produced) IGF-I of the fine flounder [31], [35]. In other fish species, there are no reports about P38/MAPK activation in muscle; thus, the present study is the first approach assessing P38 in a teleost species. A complex biology of P38, concomitant with a lack of information of this kinase in fish skeletal muscle, makes the interpretation of the data difficult. More studies are required in order to understand the role of this signal transduction in fish muscle. Meanwhile, our data suggests that P38 is not involved in the *Atrogin-1*-induced muscle atrophy as is observed in mammals.

The Akt/FoxO signaling pathway shows an opposite, inverse trend in comparison with the expression of both atrogenes. These results are in line with previous reports in both vertebrate models. In mammals, Akt activation, in addition to stimulating skeletal muscle hypertrophy, can significantly inhibit the induction of atrophy signaling [9], [33]. Genetic activation of Akt blocks atrophy-associated increases in *MuRF-1* and *Atrogin-1* transcription [33], and the mechanism by which Akt inhibits the expression of both atrogenes involves the FoxO transcription factor’s family and the upregulation of *MuRF-1* and *Atrogin-1* [9], [17], [33]. In fish, a single *in vitro* study has assessed the Akt/FoxO signaling pathway and the expression of both atrogenes, showing similar results to those in mammals [20].

The IκBα/NFκB signaling pathway is rapidly activated, concomitant with IκBα degradation during fasting, whereas during refeeding an opposite phenomenon is observed. This kinetic is highly correlated and synchronized with *MuRF-1* expression (R^2^ = 0.82, P<0.0001), but not with *Atrogin-1* (R^2^ = 0.52, P>0.05). In mammals, a linear signaling pathway conformed by IKKβ/IκBα/NFκB is sufficient to induce atrophy by stimulating the expression of *MuRF-1* but not of *Atrogin-1* [14]; which is in agreement with our results. In skeletal muscle of fish, this study constitutes the first approach linking the activation of IκBα/NFκB with the expression of *MuRF-1*.

Altogether, the activation of the IκBα/NFκB signaling pathway and upregulation of *MuRF-1,* concomitant with the inactivation of the Akt/FoxO pathway and the upregulation of *Atrogin-1,* are closely related and synchronized with an increase in ubiquitinated proteins, a previous step to protein degradation and muscle atrophy. The involvement of the ubiquitin-proteasome pathway in skeletal muscle atrophy has been well established [2], [3], [5]. Several target muscle proteins have been identified as being ubiquitinated by either MuRF-1 [44] or Atrogin-1 [44], [45], [46]. Particularly important was the discovery that the myosin heavy chain (MYH), one of the most abundant structural proteins in muscle, is a substrate of MuRF-1, thus identifying the mechanism by which MYH is depleted under atrophy conditions [47]. Interestingly, *MuRF-1* is highly expressed in muscle of the fine flounder in basal conditions and increases during fasting. The fine flounder presents a natural growth deficiency, which is reflected in limited muscle growth [31]. It could be possible to hypothesize that a high abundance of *MuRF-1* in skeletal muscle of the fine flounder might be triggering the depletion of the structural protein MYH, thus affecting muscle mass. We have previously shown that MYH expression radically deceases during fasting [Fuentes EN, Safian D, Valdes JA & Molina A. 2012, unpublished results]; however, it remains to be determined whether the atrophy in muscle of the fine flounder is due to a decrease in MYH only, or by degradation of MYH by *MuRF-1,* or both mechanisms acting concomitantly.

### Integration of the Atrophy System in Fish Skeletal Muscle

In the present study, it has been shown that the components of the atrophy system in skeletal muscle have appeared early in the evolution of vertebrates, finding some to be evolutionarily conservative (i.e. Akt/FoxO and IκBα atrogenes expression; and ubiquitination of proteins) and others as non-conservative (i.e. P38) mechanisms in comparison with higher vertebrates. Moreover, in order to better elucidate the regulation of muscle mass in this fish species, we have placed the present results into a global framework of growth in skeletal muscle by compiling the present information with our previous reports [31], [35], [48].

**Figure 8:**
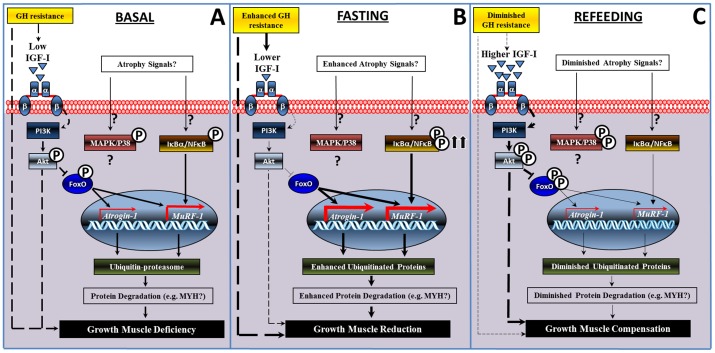
Schematic diagram illustrating the occurring events of the atrophy system in the skeletal muscle of the fine flounder. Summary of events during normal-basal (A), catabolic-fasting (B), and anabolic-refeeding (C) conditions. Unconfirmed molecules or biological processes are indicated by a question mark. (P) Denotes phosphorylation of a molecule. Dashed lines indicate previous reports. For further details see Discussion section.

In basal conditions, the fine flounder presents low muscle-derived IGF-I [31]. We have previously shown that IGF-I is able to activate the MAPK/ERK and the PI3K/Akt in muscle *in vivo* and contribute to somatic growth in this species [35]. In this context, we suggest that the basal impairment in local muscle-produced IGF-I might be responsible for the excessive expression of *MuRF-1* and high expression of *Atrogin-1*; thus, atrophying muscle and subsequently affecting somatic growth (Fig. 8A). The reason why *MuRF-1* is more expressed than *Atrogin-1* is unknown. However, considering that we did not find evidence for a P38-related expression of *Atrogin-1*, this might imply that expression of this atrogene could be controlled only by the Akt/FoxO signaling pathway. On the other hand, we did find evidence for a relationship between the IκBα/NFκB pathway and *MuRF-1*. This atrogene is also inversely related with Akt/FoxO, and thus a plausible hypothesis is that *MuRF-1* is being controlled by more extracellular stimuli and signaling pathways than *Atrogin-1* (Fig. 8A).

During fasting, local IGF-I levels decrease even further [31]. Thus, we suggest that these low levels of IGF-I may be inactivating the Akt/FoxO signaling pathway and upregulating both atrogenes, therefore enhancing protein ubiquitination. Concomitantly, the activation of the IκBα/NFκB pathway would also be increasing the transcription of *MuRF-1*, thus contributing even more to protein ubiquitination. We speculate that altogether these intracellular responses lead to muscle atrophy, diminishing somatic growth (body weight, condition factor, specific growth rate) [31], [35] (Fig. 8B).

During refeeding a rapid switch in the atrophy system is observed, particularly during short-term refeeding when local muscle-derived IGF-I increases radically [31]. We suggest that an increase in IGF-I levels may be reactivating the Akt/FoxO pathway, drastically diminishing the expression of both atrogenes and the ubiquitination of proteins (Fig. 8C). Likewise, a rapid inactivation of the IκBα/NFκB signaling pathway is observed during this period, which might also be contributing to the downregulation of *MuRF-1* (Fig. 8C). Thus, during the first stages of refeeding, strong anti-atrophy effects are observed which match with our previous observation that positive-anabolic signals exceed negative-catabolic signals [31], [35], [48] (Fig. 8C). Hence, it is possible to hypothesize that this imbalance between positive and negative signals is lay the foundation for promotion of a strong catch-up growth (body weight, condition factor, specific growth rate) during the first stages of refeeding in this species [31], [35]. Subsequently, during long-term refeeding, positive and negative signals start to become balanced, with the majority of the components returning to basal levels, as with a low production of muscle-derived IGF-I [31]. Nevertheless, during this period, we still observe that positive regulators of growth are more activated (i.e. Akt (present study), circulating IGF-I, and IGFBP-4, 5 [35], [48] while negative regulators of growth are still diminished (i.e. *Atrogin-1* (present study), and IGFBP-3, 2 [48]). Therefore, we suggest that altogether, the dynamic between these molecules would be promoting an attenuated growth, which, as a consequence, may be triggering the full-compensation of growth (body weight, condition factor, specific growth rate) in this species, as we have shown previously [28], [29] (Fig. 8C).

During refeeding a rapid switch in the atrophy system is observed, particularly during short-term refeeding when local muscle-derived IGF-I increases radically [31]. We suggest that an increase in IGF-I levels may be reactivating the Akt/FoxO pathway, drastically diminishing the expression of both atrogenes and the ubiquitination of proteins (Fig. 8C). Likewise, a rapid inactivation of the IκBα/NFκB signaling pathway is observed during this period, which might also be contributing to the downregulation of *MuRF-1* (Fig. 8C). Thus, during the first stages of refeeding, strong anti-atrophy effects are observed which match with our previous observation that positive-anabolic signals exceed negative-catabolic signals [31], [35], [48] (Fig. 8C). Hence, it is possible to hypothesize that this imbalance between positive and negative signals is lay the foundation for promotion of a strong catch-up growth (body weight, condition factor, specific growth rate) during the first stages of refeeding in this species [31], [35]. Subsequently, during long-term refeeding, positive and negative signals start to become balanced, with the majority of the components returning to basal levels, as with a low production of muscle-derived IGF-I [31]. Nevertheless, during this period, we still observe that positive regulators of growth are more activated (i.e. Akt (present study), circulating IGF-I, and IGFBP-4, 5 [35], [48] while negative regulators of growth are still diminished (i.e. *Atrogin-1* (present study), and IGFBP-3, 2 [48]). Therefore, we suggest that altogether, the dynamic between these molecules would be promoting an attenuated growth, which, as a consequence, may be triggering the full-compensation of growth (body weight, condition factor, specific growth rate) in this species, as we have shown previously [28], [29] (Fig. 8C).

## References

[1] Jagoe RT, Goldberg AL (2001) What do we really know about the ubiquitin-proteasome pathway in muscle atrophy? Curr Opin Clin Nutr Metab Care 4: 183–190. 11517350 10.1097/00075197-200105000-00003

[2] Jagoe RT, Lecker SH, Gomes M, Goldberg AL (2002) Patterns of gene expression in atrophying skeletal muscles: response to food deprivation. FASEB J 16: 1697–1712. 12409312 10.1096/fj.02-0312com

[3] Lecker SH, Jagoe RT, Gilbert A, Gomes M, Baracos V, et al (2004) Multiple types of skeletal muscle atrophy involve a common program of changes in gene expression. FASEB J 18: 39–51. 14718385 10.1096/fj.03-0610com

[4] Furuno K, Goodman MN, Goldberg AL (1990) Role of different proteolytic systems in the degradation of muscle proteins during denervation atrophy. J Biol Chem 265: 8550–8557. 2187867

[5] Tawa NE Jr, Odessey R, Goldberg AL (1997) Inhibitors of the proteasome reduce the accelerated proteolysis in atrophying rat skeletal muscles. J Clin Invest 100: 197–203. 9202072 10.1172/JCI119513 PMC508180

[6] Ciechanover A (1998) The ubiquitin–proteasome pathway: on protein death and cell life. EMBO J 17: 7151–7160. 9857172 10.1093/emboj/17.24.7151 PMC1171061

[7] Attaix D, Combaret L, Pouch MN, Taillandier D (2001) Regulation of proteolysis. Curr Opin Clin Nutr Metab Care 4: 45–49. 11122559 10.1097/00075197-200101000-00009

[8] Pickart CM (2001) Mechanisms underlying ubiquitination. Annu Rev Biochem 70: 503–533. 11395416 10.1146/annurev.biochem.70.1.503

[9] Sandri M, Sandri C, Gilbert A, Skurk C, Calabria E, et al (2004) Foxo transcription factors induce the atrophy-related ubiquitin ligase atrogin-1 and cause skeletal muscle atrophy. Cell 117: 399–412. 15109499 10.1016/s0092-8674(04)00400-3 PMC3619734

[10] Bodine SC, Latres E, Baumhueter S, Lai VK, Nunez L, et al (2001) Identification of ubiquitin ligases required for skeletal muscle atrophy. Science 294: 1704–1708. 11679633 10.1126/science.1065874

[11] Glass DJ (2003) Signalling pathways that mediate skeletal muscle hypertrophy and atrophy. Nat Cell Biol 5: 87–90. 12563267 10.1038/ncb0203-87

[12] Glass DJ (2003) Molecular mechanisms modulating muscle mass. Trends Mol Med 9: 344–350. 12928036 10.1016/s1471-4914(03)00138-2

[13] Glass DJ (2005) Skeletal muscle hypertrophy and atrophy signaling pathways. Int J Biochem Cell Biol 37: 1974–1984. 16087388 10.1016/j.biocel.2005.04.018

[14] Cai D, Frantz JD, Tawa NE, Melendez PA, Oh BC, et al (2004) IKKbeta/NF-kappaB activation causes severe muscle wasting in mice. Cell 119: 285–298. 15479644 10.1016/j.cell.2004.09.027

[15] Li YP, Chen YL, John J (2005) TNF-alpha acts via p38 MAPK to stimulate expression of the ubiquitin ligase atrogin1/MAFbx in skeletal muscle. FASEB J 19: 362–370. 15746179 10.1096/fj.04-2364com PMC3099533

[16] Moylan JS, Smith JD, Chamber MA, McLoughlin TJ, Reid MB (2008) TNF induction of atrogin-1/MAFbx mRNA depends on Foxo4 expression but not AKT-Foxo1/3 signaling, Am J Physiol Cell Physiol. 295: C986–C993. 10.1152/ajpcell.00041.2008 PMC2575831 18701653

[17] Kamei Y, Miura S, Suzuki M, Kai Y, Mizukami J, et al (2004) Skeletal muscle FOXO1 (FKHR) transgenic mice have less skeletal muscle mass, down-regulated Type I (slow twitch/red muscle) fiber genes, and impaired glycemic control. J Biol Chem 27: 41114–41123. 10.1074/jbc.M400674200 15272020

[18] Bower NI, de la Serrana DG, Johnston IA (2010) Characterization and differential regulation of MAFbx/Atrogin-1 alpha and beta transcripts in skeletal muscle of Atlantic salmon (*Salmo salar*). Biochem Biophys Res Commun 396: 265–271. 20399749 10.1016/j.bbrc.2010.04.076

[19] Tacchi L, Bickerdike R, Secombes CJ, Pooley NJ, Urquhart KL, et al (2010) Ubiquitin E3 ligase atrogin-1 (Fbox-32) in Atlantic salmon (Salmo salar): sequence analysis, genomic structure and modulation of expression. Comp Biochem Physiol B Biochem Mol Biol 157: 364–373. 20728561 10.1016/j.cbpb.2010.08.004

[20] Cleveland BM, Weber GM (2010) Effects of insulin-like growth factor-I, insulin, and leucine on protein turnover and ubiquitin ligase expression in rainbow trout primary myocytes. Am J Physiol Regul Integr Comp Physiol 298: R341–350. 20007517 10.1152/ajpregu.00516.2009

[21] Cleveland BM, Evenhuis JP (2010) Molecular characterization of atrogin-1/F-box protein-32 (FBXO32) and F-box protein-25 (FBXO25) in rainbow trout (Oncorhynchus mykiss): Expression across tissues in response to feed deprivation. Comp Biochem Physiol B Biochem Mol Biol 157: 248–257. 20601059 10.1016/j.cbpb.2010.06.010

[22] Seiliez I, Panserat S, Skiba-Cassy S, Fricot A, Vachot C, et al (2008) Feeding status regulates the polyubiquitination step of the ubiquitin-proteasome-dependent proteolysis in rainbow trout (*Oncorhynchus mykiss*) muscle. J Nutr 138: 487–491. 18287354 10.1093/jn/138.3.487

[23] Salem M, Kenney PB, Rexroad CE III, Yao J (2006) Microarray gene expression analysis in atrophying rainbow trout muscle: a unique nonmammalian muscle degradation model. Physiol Genomics 28: 33–45. 16882886 10.1152/physiolgenomics.00114.2006

[24] Lin YC, Chiu KH, Shiea J, Huang HW, Mok HK (2011) Seasonal changes in atrophy-associated proteins of the sonic muscle in the big-snout croaker, Johnius macrorhynus (Pisces, Sciaenidae), identified by using a proteomic approach. Fish Physiol Biochem 37: 977–991. 21553060 10.1007/s10695-011-9502-3

[25] Salem M, Kenney PB, Rexroad CE III, Yao J (2010) Proteomic signature of muscle atrophy in rainbow trout. J Proteomics 73: 778–789. 19903543 10.1016/j.jprot.2009.10.014

[26] Johnston IA (1982) Physiology of muscle in hatchery raised fish. Comp Biochem Physiol B Biochem Mol Biol 73 105–124: 23.

[27] Johnston IA (1999) Muscle development and growth: potential implications for flesh quality in fish. Aquaculture 177: 99–115.

[28] Stickland NC (1983) Growth and development of muscle fibres in the rainbow trout *Salmo gairdneri.* . J Anat 137: 323–333. 6630043 PMC1171824

[29] Weatherley AH, Gill HS, Lobo AF (1988) Recruitment and maximal diameter of axial muscle fibres in teleosts and their relationship to somatic muscle growth and ultimate size. J Fish Biol 33: 851–859.

[30] Fuentes EN, Kling P, Einarsdottir IE, Alvarez M, Valdés JA, et al (2012) Plasma leptin and growth hormone levels in the fine flounder (*Paralichthys adspersus*) increase gradually during fasting and decline rapidly after refeeding. Gen Comp Endocrinol. 177: 120–127. 10.1016/j.ygcen.2012.02.019 22429729

[31] Fuentes EN, Einarsdottir IE, Valdes JA, Alvarez M, Molina A, et al (2012) Inherent growth hormone resistance in the skeletal muscle of the fine flounder is modulated by nutritional status and is characterized by high contents of truncated GHR, impairment in the JAK2/STAT5 signaling pathway, and low IGF-I expression. Endocrinology. 153: 283–294. 10.1210/en.2011-1313 22028448

[32] Latres E, Amini AR, Amini AA, Griffiths J, Martin FJ, et al (2005) Insulin-like growth factor-1 (IGF-1) inversely regulates atrophy-induced genes via the phosphatidylinositol 3-kinase/Akt/mammalian target of rapamycin (PI3K/Akt/mTOR) pathway. J Biol Chem 280: 2737–2744. 15550386 10.1074/jbc.M407517200

[33] Stitt TN, Drujan D, Clarke BA, Panaro F, Timofeyva Y, et al (2004) The IGF-1/PI3K/Akt pathway prevents expression of muscle atrophy-induced ubiquitin ligases by inhibiting FOXO transcription factors. Mol Cell 14: 395–403. 15125842 10.1016/s1097-2765(04)00211-4

[34] Sacheck JM, Ohtsuka A, McLary SC, Goldberg AL (2004) IGF-1 stimulates muscle growth by suppressing protein breakdown and expression of atrophy-related ubiquitin-ligases, atrogin-1 and MuRF1. Am J Physiol Endocrinol Metab. 287: E591–E601. 10.1152/ajpendo.00073.2004 15100091

[35] Fuentes EN, Björnsson BT, Valdés JA, Einarsdottir IE, Lorca B, et al (2011) IGF-I/PI3K/Akt and IGF-I/MAPK/ERK pathways in vivo in skeletal muscle are regulated by nutrition and contribute to somatic growth in the fine flounder. Am J Physiol Regul Integr Comp Physiol 300: R1532–1542. 21389330 10.1152/ajpregu.00535.2010

[36] Bower NI, Johnston IA (2010) Transcriptional regulation of the IGF signaling pathway by amino acids and insulin-like growth factors during myogenesis in Atlantic salmon. PLoS One 5: e11100. 20559434 10.1371/journal.pone.0011100 PMC2885424

[37] Bustin SA, Benes V, Garson JA, Hellemans J, Huggett J, et al (2009) The MIQE guidelines: minimum information for publication of quantitative real-time PCR experiments. Clin Chem 55: 611–622. 19246619 10.1373/clinchem.2008.112797

[38] Vandesompele J, De Preter K, Pattyn K, Poppe B, Van Roy N, et al (2002) Accurate normalization of real-time quantitative RT-PCR data by geometric averaging of multiple internal control genes. Genome Biol 3 RESEARCH0034: 32. 10.1186/gb-2002-3-7-research0034 PMC126239 12184808

[39] Caraux G, Pinloche S (2005) PermutMatrix: a graphical environment to arrange gene expression profiles in optimal linear order. Bioinformatics 21 1280–1281: 33. 10.1093/bioinformatics/bti141 15546938

[40] Lluís F, Perdiguero E, Nebreda AR, Muñoz-Cánoves P (2006) Regulation of skeletal muscle gene expression by p38 MAP kinases. Trends Cell Biol 16: 36–44. 16325404 10.1016/j.tcb.2005.11.002

[41] Zhang G, Jin B, Li YP (2011) C/EBPβ mediates tumour-induced ubiquitin ligase atrogin1/MAFbx upregulation and muscle wasting. EMBO J 30: 4323–4335. 21847090 10.1038/emboj.2011.292 PMC3199382

[42] Trendelenburg AU, Meyer A, Jacobi C, Feige JN, Glass DJ (2012) TAK-1/p38/nNFκB signaling inhibits myoblast differentiation by increasing levels of Activin A. Skelet Muscle. 2: 3. 10.1186/2044-5040-2-3 PMC3295657 22313861

[43] Ren H, Accili D, Duan C (2010) Hypoxia converts the myogenic action of insulin-like growth factors into mitogenic action by differentially regulating multiple signaling pathways. Proc Natl Acad Sci USA 107: 5857–5862. 20231451 10.1073/pnas.0909570107 PMC2851893

[44] Witt SH, Granzier H, Witt CC, Labeit S (2005) MURF-1 and MURF-2 target a specific subset of myofibrillar proteins redundantly: towards understanding MURF-dependent muscle ubiquitination. J Mol Biol 350: 713–722. 15967462 10.1016/j.jmb.2005.05.021

[45] Lagirand-Cantaloube J, Cornille K, Csibi A, Batonnet-Pichon S, Leibovitch MP, et al (2009) Inhibition of atrogin-1/MAFbx mediated MyoD proteolysis prevents skeletal muscle atrophy in vivo. PLoS One 4: e4973. 19319192 10.1371/journal.pone.0004973 PMC2656614

[46] Lagirand-Cantaloube J, Offner N, Csibi A, Leibovitch MP, Batonnet-Pichon S, et al (2008) The initiation factor eIF3-f is a major target for atrogin1/MAFbx function in skeletal muscle atrophy. EMBO J 27: 1266–1276. 18354498 10.1038/emboj.2008.52 PMC2367397

[47] Clarke BA, Drujan D, Willis MS, Murphy LO, Corpina RA, et al (2007) The E3 Ligase MuRF1 degrades myosin heavy chain protein in dexamethasone-treated skeletal muscle. Cell Metab 6: 376–385. 17983583 10.1016/j.cmet.2007.09.009

[48] Safian D, Fuentes EN, Valdés JA, Molina A (2012) Dynamic transcriptional regulation of autocrine/paracrine igfbp1, 2, 3, 4, 5, and 6 in the skeletal muscle of the fine flounder during different nutritional statuses. J Endocrinol 214: 95–108. 22499735 10.1530/JOE-12-0057

