# Correction: Catabolic Signaling Pathways, Atrogenes, and Ubiquitinated Proteins Are Regulated by the Nutritional Status in the Muscle of the Fine Flounder

**DOI:** 10.1371/journal.pone.0244410

**Published:** 2020-12-28

**Authors:** Eduardo N. Fuentes, Pamela Ruiz, Juan Antonio Valdes, Alfredo Molina

After this article [1] was published, concerns were raised about Fig 6.

Specifically,

- In the ‘Free Ubiquitin’ panels, lanes 3–6 in Fig 6A appear similar to lanes 1–4 in Fig 6B.
- In the ‘Total proteins’ panels, lanes 5–8 in Fig 6A appear similar to lanes 1–4 in Fig 6B.

**Fig 6:**
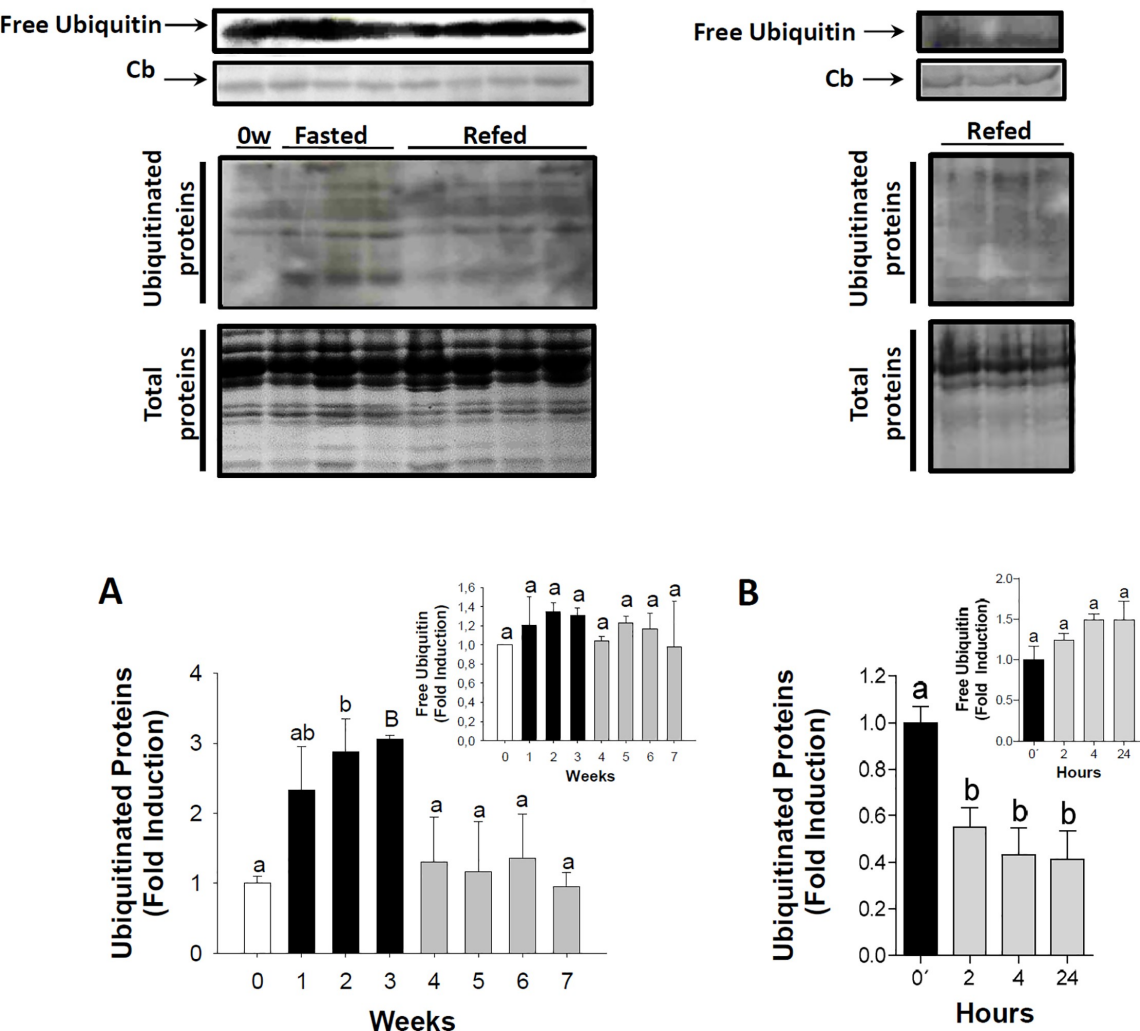
Ubiquitination of proteins in the skeletal muscle during long-term fasting and refeeding and short-term refeeding. Ubiquitinated protein during long-term fasting and refeeding (A) and short-term refeeding (B). Inserts in A and B show the amount of free ubiquitin during the trial. Percentage of ubiquitinated proteins during long-term fasting and refeeding and short-term refeeding are shown in graphs below the image data. These data were normalized to total protein levels. White, black and grey bars represent periods of feeding, fasting and refeeding, respectively. A probability level of P<0.05 (lower case letters) and P<0.01 (by upper case letters) was used to indicate statistical significances. Results are expressed as means±SEM (n  =  3). Different letters indicate significant differences among sampling points of each group, respectively. Abbreviations: Cb  =  *Coomassie* blue staining; 0′  =  zero hour of short-term refeeding corresponding to the end of fasting period (week 3).

The authors noted that these issues resulted from errors in preparing Fig 6B. The authors apologize for these errors, confirm that the original published Fig 6A correctly reported the indicated results, and provide here an updated Fig 6 in which the indicated data from the original experiment are reported in Fig 6B. Underlying data supporting Fig 6 are in S1 File and S2 File. The raw quantitative data underlying Fig 6A are no longer available; S2 File and the graphs in the updated version of Fig 6B report results of re-quantification done after the article was published due to the unavailability of the original data.

The 3-week data for the long-term fasting and re-feeding experiment (lane 4 in Fig 6A) were used as the 0 timepoint control when quantifying results for the short-term re-feeding experiment reported in Fig 6B. These data correspond to the same experimental replicate, but the long-term fasting and re-feeding data were obtained on a different blot than the short-term re-feeding data. Consequently, conclusions cannot be drawn based on comparisons of the 3-week long-term fasting and re-feeding data versus the 2, 4, and 24 hour short-term re-feeding data. In light of this issue, the results and conclusions statements about ubiquitination during short-term re-feeding are not supported.

The original data underlying several results reported in the article’s figures are no longer available. The unavailable data include quantitative data underlying results reported in Figs 2–4 and some results in Fig 7, as well as image data underlying select panels of Figs 1 and 3. Data underlying other results in the article are available upon request from the corresponding author.

## Supporting information

S1 File [Raw data Fig 6 ALL FIGURES.pdf]. Raw image data underlying blot and gel images shown in Fig 6. The revised figure includes data from replica 3 (pages 5–8 of the PDF).
(PDF)

S2 File [Ubiquitin WB analysis plos.xls]. Quantitative data to support the revised version of Fig 6B.
(XLSX)
