# Supplementary material for: Correction: Catabolic Signaling Pathways, Atrogenes, and Ubiquitinated Proteins Are Regulated by the Nutritional Status in the Muscle of the Fine Flounder
Source: PLoS One. 2020 Dec 28;15(12):e0244410. doi: 10.1371/journal.pone.0244410 (PMC7769450; doi:10.1371/journal.pone.0244410)
Supplement: S1 File — Raw image data underlying blot and gel images shown in Fig 6. The revised figure includes data from replica 3 (pages 5–8 of the PDF). (PDF) [file pone.0244410.s001.pdf]

Biological replica number one, fish one (N = 1)
Ubiquitinated Proteins Blot long-term fasting and refeeding

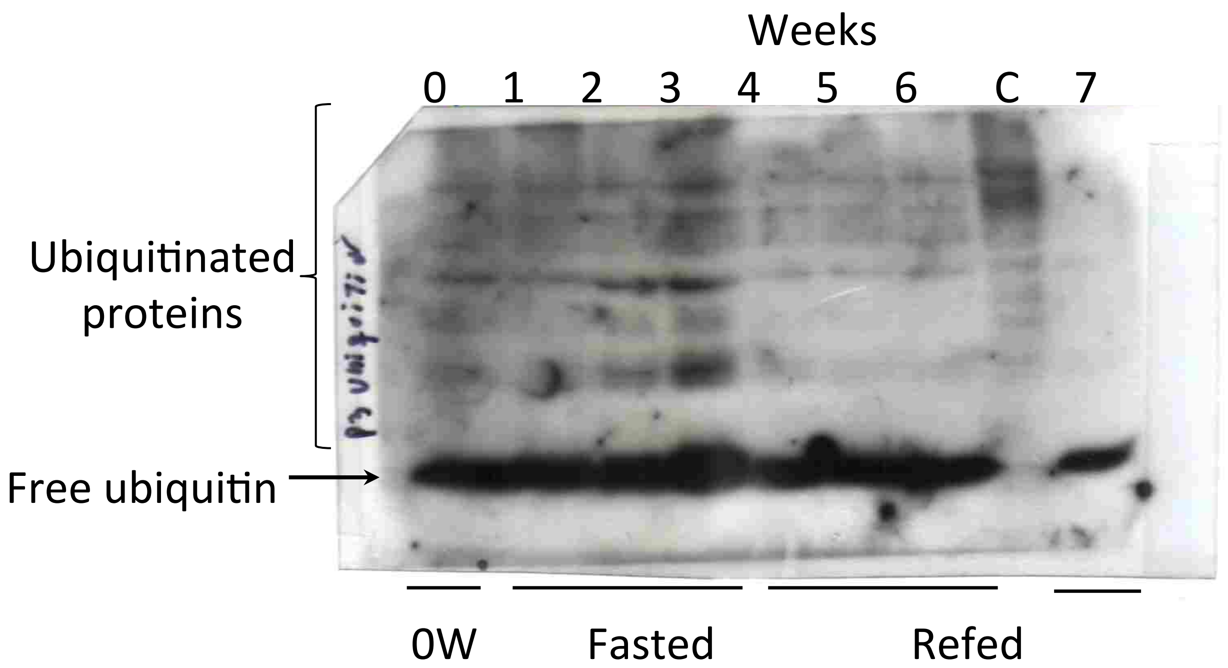

Note 1: C = Control for antibody specificity. As stated in Material and Methods section, the antibodies use in this study are commercial antibodies developed against mammalian epitopes, therefore primary culture of rat muscle was used as control for antibody specificity.

Note 2: There was a mistake in loading the SDS-gel. However, this mistake in the order of the loading has no impact on the results and quantification due that point seven (7 weeks) was the line that was used for quantification.

Biological replica number 1, fish one (N = 1)
Total Proteins SDS-gel long-term fasting and refeeding

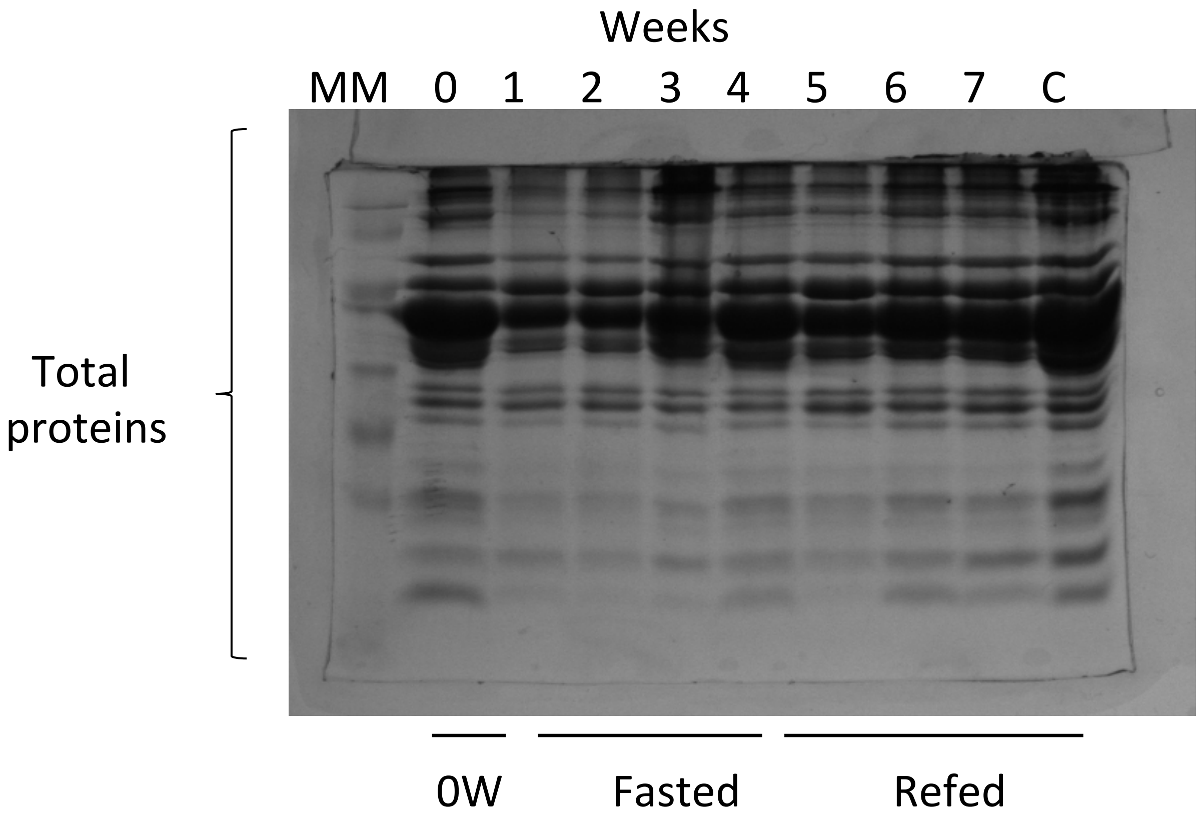

NOTE 1: C = Loading control (primary culture of rat muscle); MM = Molecular marker. This SDS-gel was an independent run gel. The amounts of protein loaded are the same that the gels used for the Western blot.

Biological replica number two, fish two (N = 2)
Ubiquitinated Proteins Blot long-term fasting and refeeding

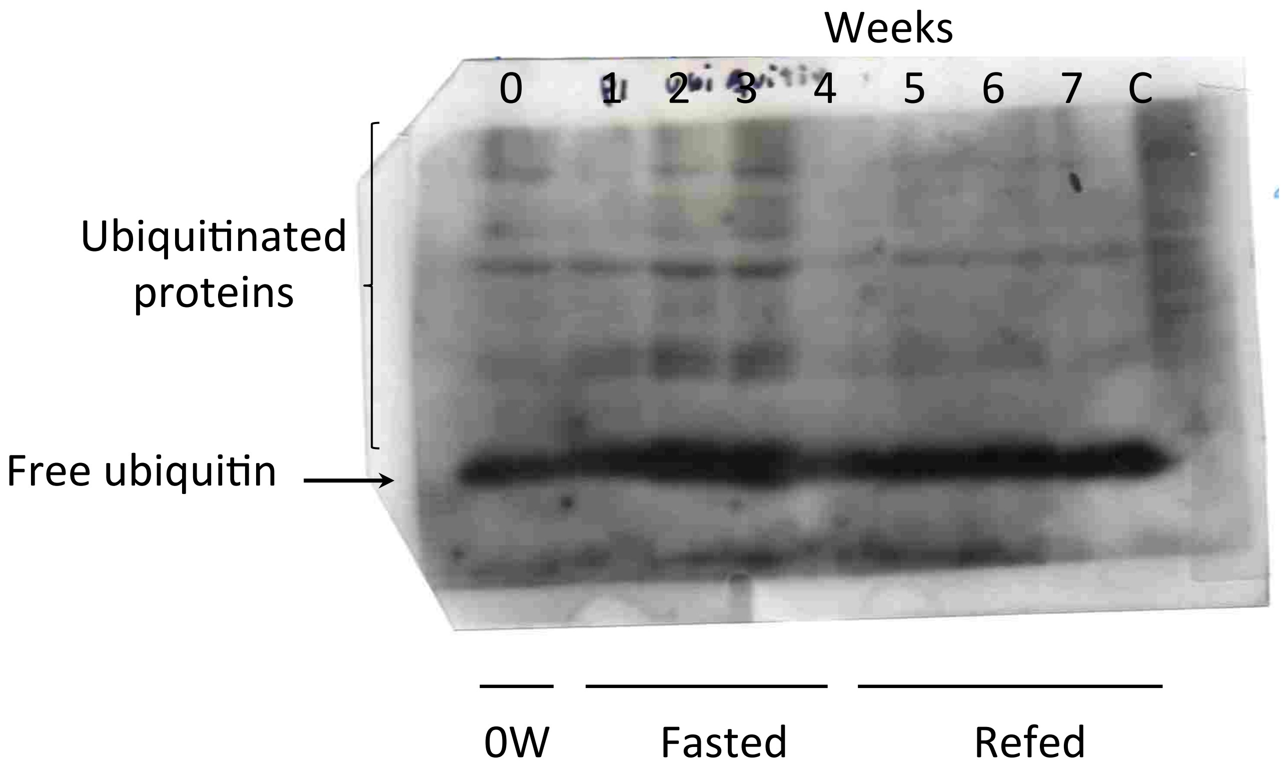

Note 1: C = Control for antibody specificity. As stated in Material and Methods section, the antibodies use in this study are commercial antibodies developed against mammalian epitopes, therefore primary culture of rat muscle was used as control for antibody specificity.

Biological replica number two, fish two (N = 2)
Total Proteins SDS-gel long-term fasting and refeeding

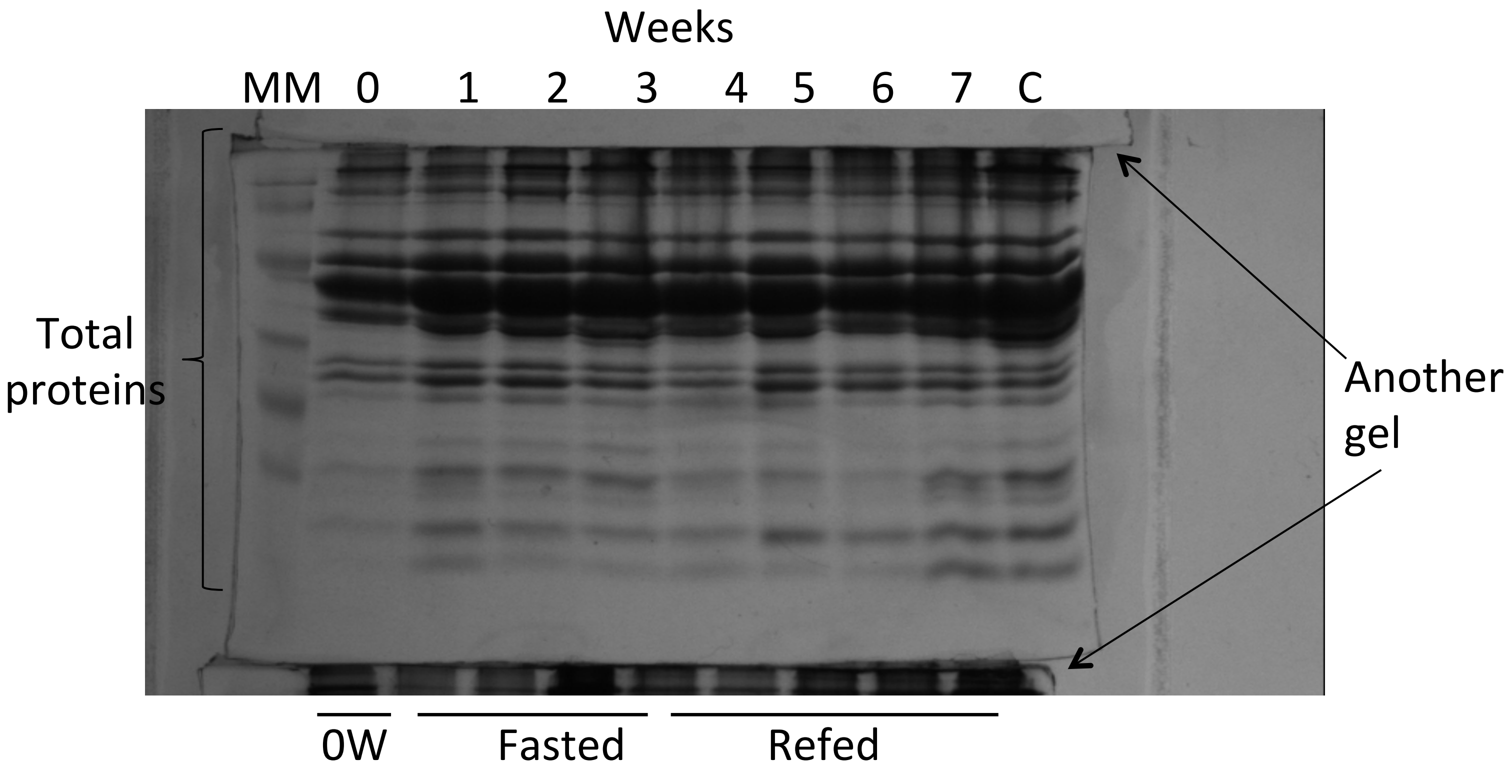

NOTE 1: C = Loading control (primary culture of rat muscle); MM = Molecular marker. This SDS-gel was an independent run gel. The amounts of protein loaded are the same that the gels used for the Western blot.

Biological replica number three, fish three (N = 3)
Ubiquitinated Proteins Blot long-term fasting and refeeding

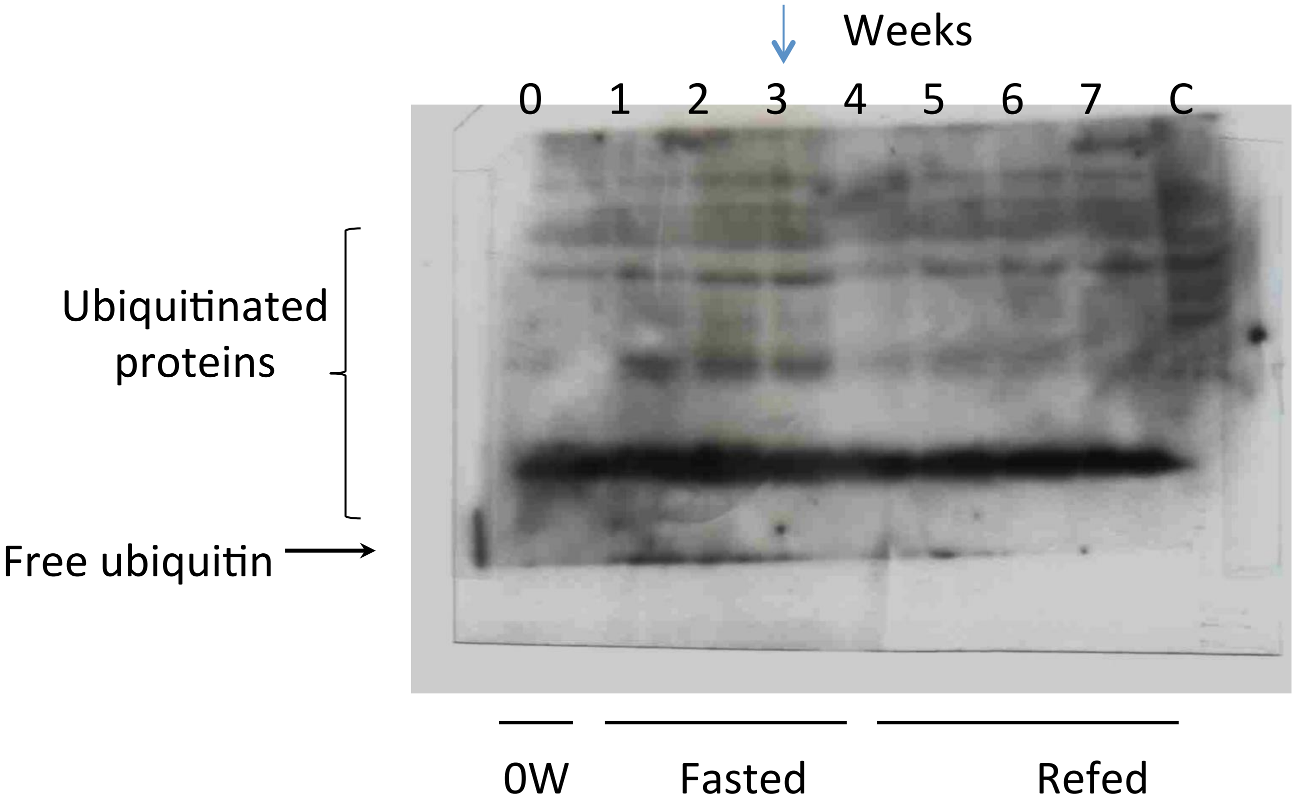

NOTE 1: This image corresponds to the representative blot shown in the PLOS ONE publication (labeled as Ubiquitinated proteins and free ubiquitin respectively in Fig. 6). 3 weeks (arrow) correspond to the to the cropped representative image shown in the new Fig. 6.

Note 2: C = Control for antibody specificity. As stated in Material and Methods section, the antibodies use in this study are commercial antibodies developed against mammalian epitopes, therefore primary culture of rat muscle was used an antibody control .

Biological replica number three, fish three (N = 3)
Total Proteins SDS-gel long-term fasting and refeeding

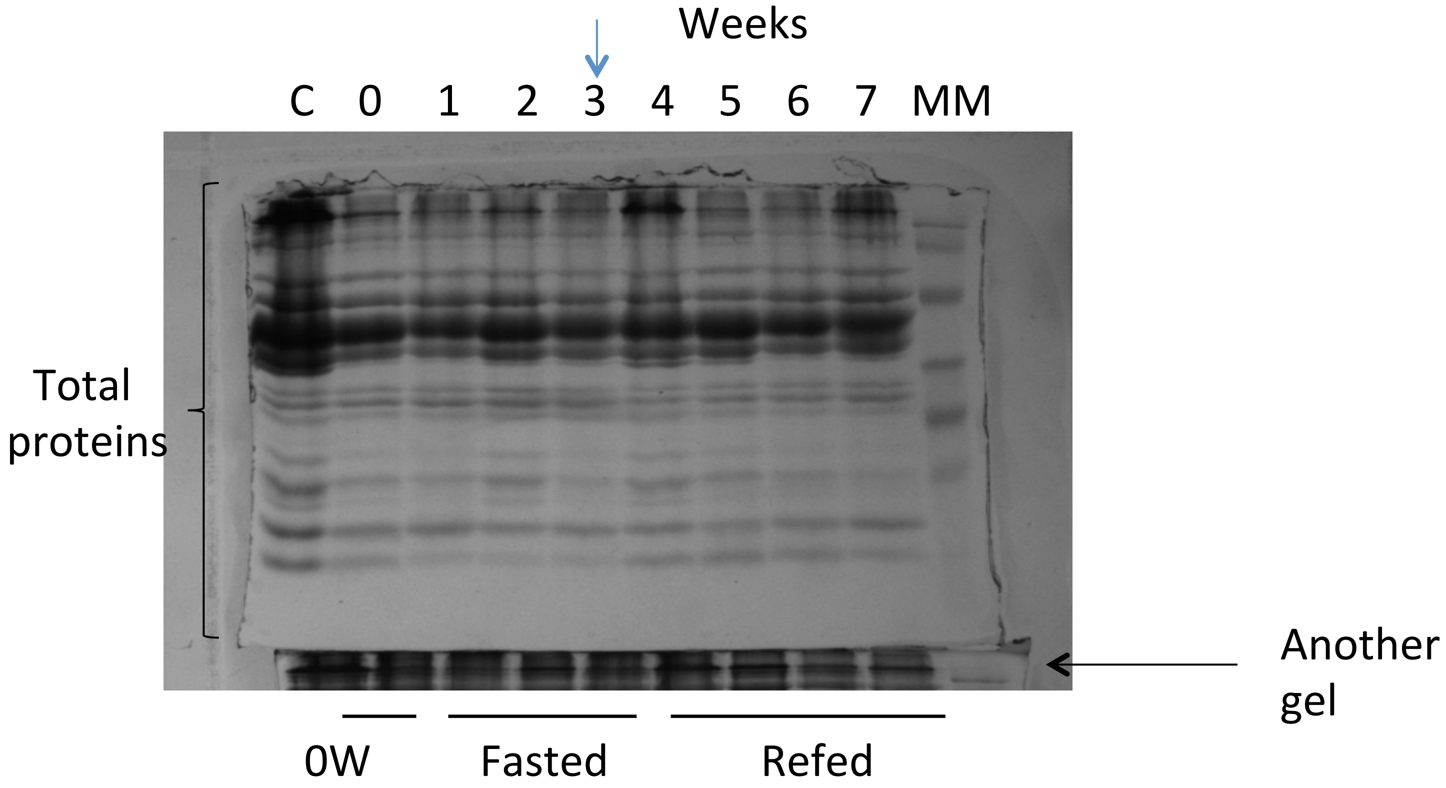

NOTE 1: This image corresponds to the representative SDS gel shown in the PLOS ONE publication (labeled as total proteins in Fig. 6). 3 weeks (arrow) correspond to the to the cropped representative image shown in the new Fig. 6.

NOTE 2: C = Loading control (primary culture of rat muscle); MM = Molecular Marker. This SDS-gel was an independent rungel. The amounts of protein loaded are the same that the gels used for the Western blot.

Biological replica number one, two, and three, fish one, two, and three (N = 1, 2, 3)

Ubiquitinated proteins blot short-term refeeding

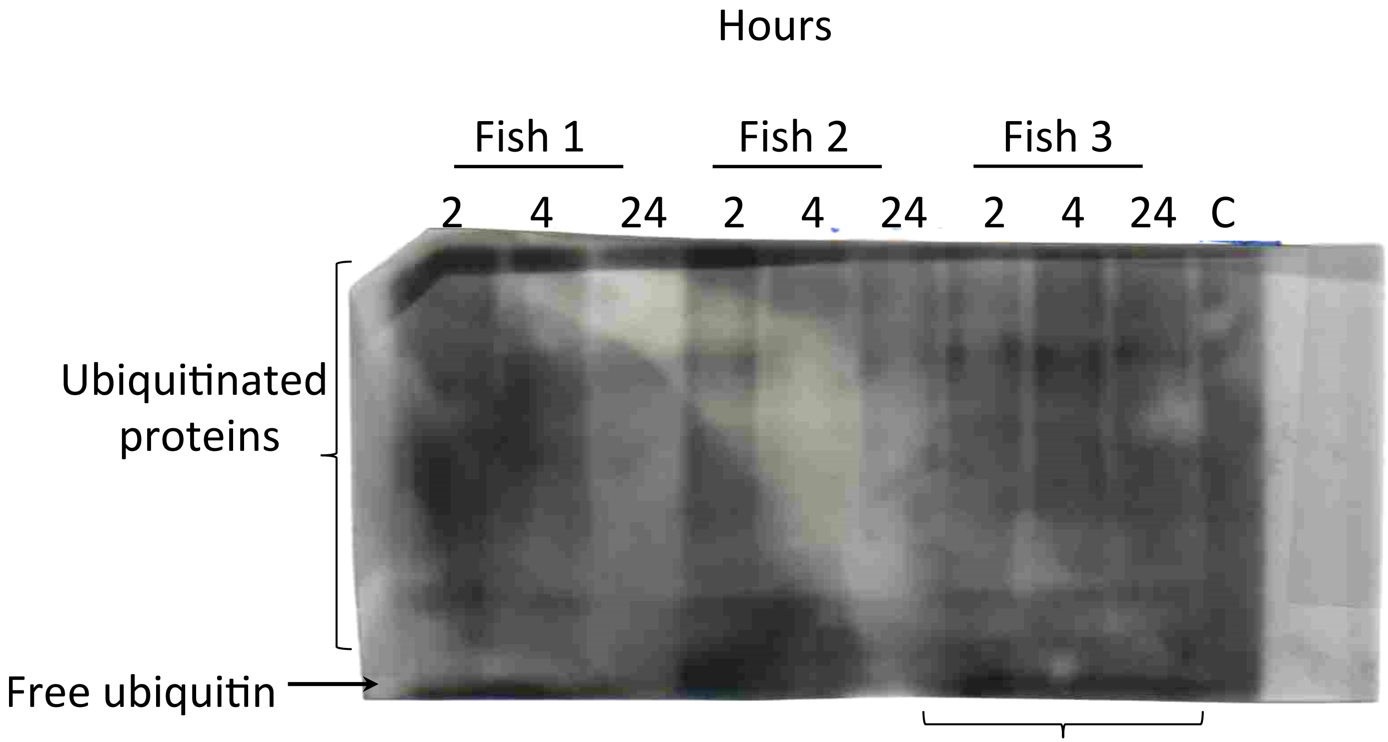

This area of the blot correspond to the cropped representative image shown in the new Fig. 6 (labeled as ubiquitinated proteins and free ubiquitin respectively in Fig. 6). This area correspond to the biological replica number 3

Biological replica number one, two, and three, fish one, two, and three (N = 1, 2, 3)
Total proteins SDS-gel short-term refeeding

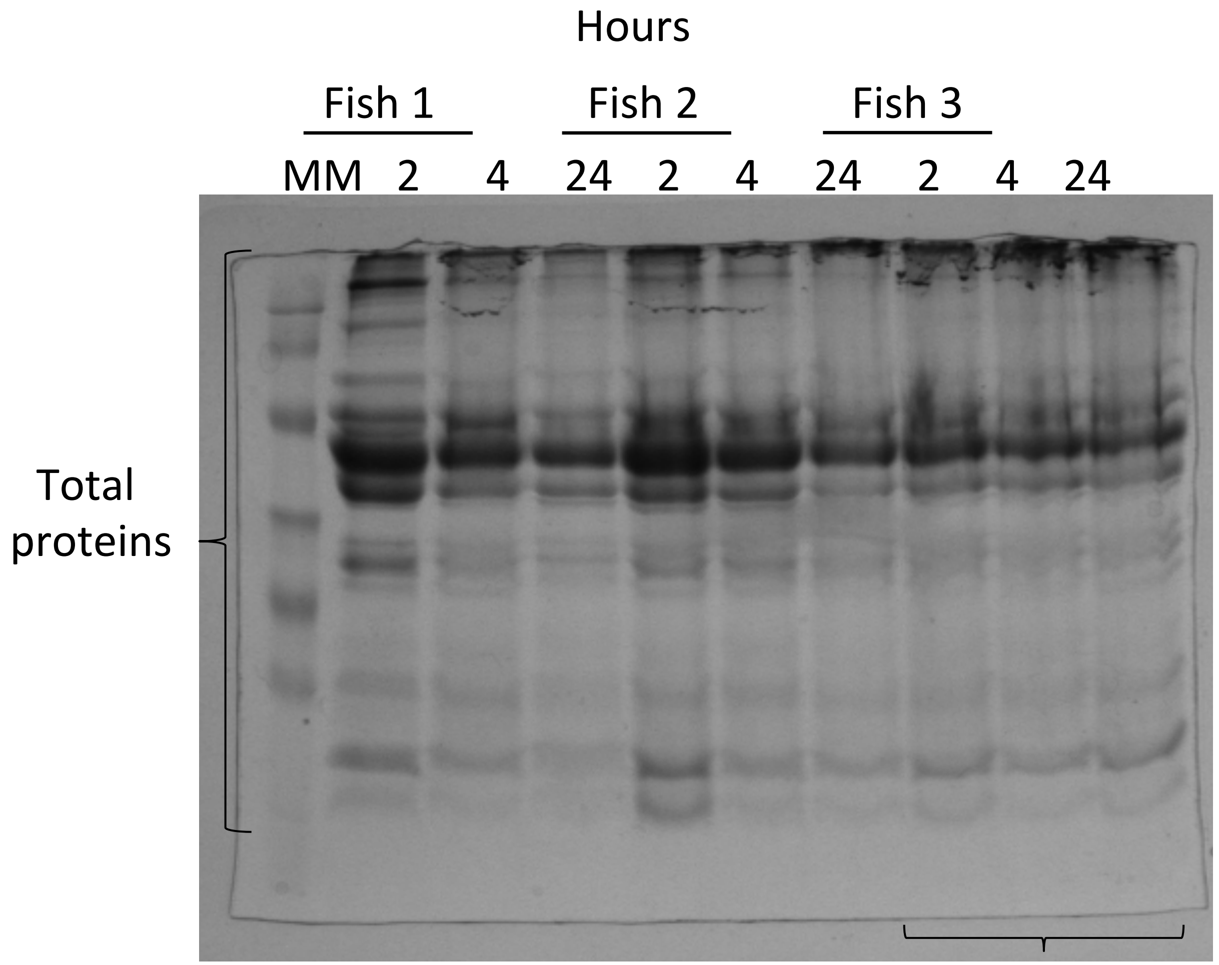

This area of the gel correspond to the cropped representative image shown in the new Fig. 6 (labeled as total proteins in Fig. 6). This area correspond to the biological replica number 3
